# Analysis of Pigment-Dispersing Factor Neuropeptides and Their Receptor in a Velvet Worm

**DOI:** 10.3389/fendo.2020.00273

**Published:** 2020-05-12

**Authors:** Christine Martin, Lars Hering, Niklas Metzendorf, Sarah Hormann, Sonja Kasten, Sonja Fuhrmann, Achim Werckenthin, Friedrich W. Herberg, Monika Stengl, Georg Mayer

**Affiliations:** ^1^Department of Zoology, Institute of Biology, University of Kassel, Kassel, Germany; ^2^Department of Animal Physiology, Institute of Biology, University of Kassel, Kassel, Germany; ^3^Department of Biochemistry, Institute of Biology, University of Kassel, Kassel, Germany

**Keywords:** PDF, PDFR, BRET, Epac, *E. rowelli*, Onychophora, Panarthropoda, Ecdysozoa

## Abstract

Pigment-dispersing factor neuropeptides (PDFs) occur in a wide range of protostomes including ecdysozoans (= molting animals) and lophotrochozoans (mollusks, annelids, flatworms, and allies). Studies in insects revealed that PDFs play a role as coupling factors of circadian pacemaker cells, thereby controlling rest-activity rhythms. While the last common ancestor of protostomes most likely possessed only one *pdf* gene, two *pdf* homologs, *pdf-I* and *pdf-II*, might have been present in the last common ancestors of Ecdysozoa and Panarthropoda (Onychophora + Tardigrada + Arthropoda). One of these homologs, however, was subsequently lost in the tardigrade and arthropod lineages followed by independent duplications of *pdf-I* in tardigrades and decapod crustaceans. Due to the ancestral set of two *pdf* genes, the study of PDFs and their receptor (PDFR) in Onychophora might reveal the ancient organization and function of the PDF/PDFR system in panarthropods. Therefore, we deorphanized the PDF receptor and generated specific antibodies to localize the two PDF peptides and their receptor in the onychophoran *Euperipatoides rowelli*. We further conducted bioluminescence resonance energy transfer (BRET) experiments on cultured human cells (HEK293T) using an Epac-based sensor (Epac-L) to examine cAMP responses in transfected cells and to reveal potential differences in the interaction of PDF-I and PDF-II with PDFR from *E. rowelli*. These data show that PDF-II has a tenfold higher potency than PDF-I as an activating ligand. Double immunolabeling revealed that both peptides are co-expressed in *E. rowelli* but their respective levels of expression differ between specific cells: some neurons express the same amount of both peptides, while others exhibit higher levels of either PDF-I or PDF-II. The detection of the onychophoran PDF receptor in cells that additionally express the two PDF peptides suggests autoreception, whereas spatial separation of PDFR- and PDF-expressing cells supports hormonal release of PDF into the hemolymph. This suggests a dual role of PDF peptides—as hormones and as neurotransmitters/neuromodulators—in Onychophora.

## Introduction

Pigment-dispersing factors (PDFs) and pigment-dispersing hormones (PDHs) are neuropeptides that occur in various protostomes and have been mainly studied in nematodes, crustaceans and insects [reviewed in (1, 2)]. In decapod crustaceans, PDH controls a circadian relocation of the retinal pigment to shield the photoreceptor cells from light and also orchestrates circadian migrations of pigment within the integumental chromatophores (3–6). The crustacean PDHs, however, seem to not only play a hormonal role in mediating pigment dispersion but might additionally act as neurotransmitters or neuromodulators in the nervous system (7–12). The same holds true for the insect PDFs—homologs of the crustacean PDHs (4, 13). PDFs are synthesized by the circadian clock neurons in various insect species and are involved in different aspects of circadian timing (1, 14–34).

**Figure 1:**
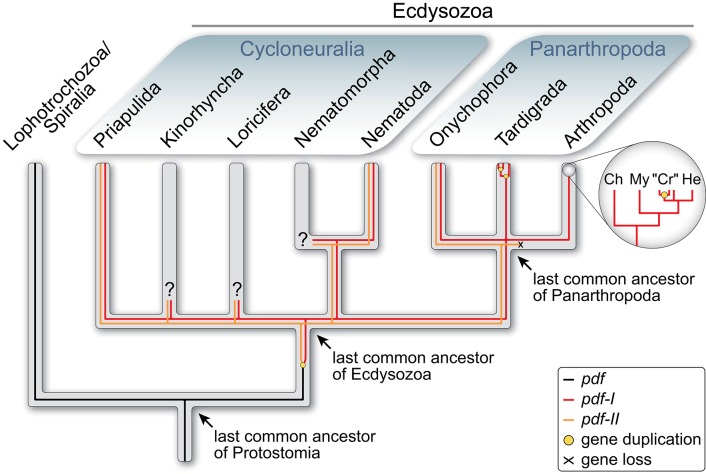
Evolutionary history of *pdf/pdh* genes in Ecdysozoa [modified from Mayer et al. (38)]. Phylogenetic relationship of Ecdysozoa from Giribet and Edgecombe (40). Two *pdf/pdh* genes were present in the last common ancestor of Ecdysozoa and have been retained at least in Priapulida, Nematoda and Onychophora, whereas *pdf-II* was lost in Tardigrada and Arthropoda. Subsequent gene duplication occurred twice in tardigrades and at least once in crustaceans. Ch, Chelicerata; “Cr”, “Crustacea”; He, Hexapoda; My, Myriapoda.

The PDFs of the fruit fly *Drosophila melanogaster* and the cockroach *Rhyparobia maderae* are analogs of the vasoactive intestinal peptide (VIP) of mammals (35). It must be noted, however, that despite their similar function in the circadian pacemaker systems, the insect PDFs (and crustacean PDHs) do not share a common ancestry with VIP. The ancestral *pdf* gene rather evolved in the protostome lineage, as it occurs in both spiralians and ecdysozoans but not in deuterostomes (36–38). The report of potential PDF precursors in echinoderms and an enteropneust (39) should be taken with caution due to the low sequence similarity with the PDFs/PDHs of insects and crustaceans [cf. Figure 7 in (39)] and the lack of a phylogenetic analysis. Interestingly, while the last common ancestor of protostomes possessed only one *pdf* gene, which has been retained at least in mollusks and annelids [(36, 37); cf. supporting information Supplementary Figures 1 and 2 in (38)], a duplication of this gene might have occurred in the ecdysozoan lineage (Figure 1). Two homologs, *pdf-I* and *pdf-II*, have been identified thus far in priapulids, nematodes and onychophorans (38, 41, 42), whereas *pdf-II* seems to have been subsequently lost in tardigrades and arthropods. Even more intriguing is the independent duplication of the retained *pdf-I* gene in tardigrades, which show three in-paralogs, and decapod crustaceans, which express two to three PDH isoforms but seem to possess only two *pdh* genes (11, 38, 43–48).

Since two PDF peptides were most likely encoded in the genome of the last common ancestor of Ecdysozoa (Figure 1), the question arises of whether their ancestral role was in light-dependent pigment dispersion, in coupling of circadian clocks or in other processes. Alternatively, these peptides might have played divergent or multiple roles. Research on nematodes revealed that their two *pdf* genes express three PDFs that most likely also have an impact on circadian timing (49). There, the same peptides serve several functions including the control of locomotion, mate searching, mechano- and chemosensation as well as the sensation of oxygen (41, 50). Accordingly, in the nematode nervous system PDFs occur in nearly all sensory neurons that also control mate searching and locomotor activity patterns (42). Insects instead possess fewer PDF-expressing cells organized in 2–4 clusters associated with each compound eye and few additional somata showing a species-specific distribution in the protocerebrum [e.g., (14–16, 22, 23, 25, 29, 30, 51–56)]. The situation in crustaceans seems to be more complex, but they also possess PDH-immunoreactive somata in the median protocerebrum, in addition to those associated with the eye stalks (7, 8, 11).

Similar to crustaceans, Mayer et al. (38) detected numerous immunoreactive somata in the median protocerebrum of six distantly related onychophoran species using a broadly reactive antiserum raised against the synthetic β-PDH peptide of the crustacean *Uca pugilator* (7). These and other results indicate a broad and elaborate distribution of PDFs in the peripheral and central nervous system of onychophorans. However, since the applied antiserum recognized both onychophoran peptides, Ony-PDF-I and Ony-PDF-II, despite its higher affinity to Ony-PDF-I [83.3% sequence similarity as opposed by 55.6–61.1% to Ony-PDF-II; cf. Figure 1 in (38)], it remains unclear whether these two peptides are co-expressed or show distinct distributions in various tissues and cells. To clarify this issue, we obtained specific antibodies against each PDF peptide of the onychophoran *Euperipatoides rowelli* (Peripatopsidae) and performed double immunolabeling. The gathered data provide insights into the ancestral distribution and potential roles of the two PDF peptides in the last common ancestors of Panarthropoda and Ecdysozoa.

Another important goal of our study was to deorphanize and immunolocalize the PDF receptor (PDFR) of onychophorans. Investigations of PDFRs are generally scarce. These membrane proteins belong to the ancient family of G protein-coupled receptors (GPCRs) and have a similar function to the VPAC_2_ receptors in the circadian system of mammals [reviewed in (35)]. However, while PDFRs are activated by PDF/PDH peptides, the VPAC_2_ receptors rather bind the aforementioned vasoactive intestinal peptide (VIP). Despite their important functions, only little is known about the origin and evolutionary history of PDFRs and VPAC_2_ receptors.

The PDFR was initially deorphanized in the fruit fly *D. melanogaster* by three simultaneous studies: one of them examined a range of GPCRs for their sensitivity to PDF (57) and the other two reported that mutants lacking the *groom-of-PDF* (*gop*) or the *han* gene (referred to as *pdfr* thereafter) imitate phenotypical behavior of *pdf* null mutants (58, 59). Subsequently, PDFR was shown to be expressed in 60% of pacemaker neurons (60), a few additional, clock-unrelated neurons as well as within the visual system of the fruit fly (61). Like *D. melanogaster*, the nematode *Caenorhabditis elegans* possesses one *pdfr* gene but three PDFR splice variants (41), all of which show a dose-dependent sensitivity to the three nematode PDFs (PDF-1a, PDF-1b, and PDF-2). The nematode PDFRs have been localized in the motor neurons of the nervous system as well as in nearly all muscle cells (41). Apart from *D. melanogaster* and *C. elegans*, to our knowledge there are no immunohistochemical data on the local distribution of PDFRs in other animals.

To improve our understanding of the PDF/PDFR system in onychophorans, we screened the transcriptome of *E. rowelli* (62) for GPCR genes and identified a single *pdfr* candidate. We further performed functional analyses using synthetic PDF-I and PDF-II peptides (based on sequences from *E. rowelli*) in conjunction with bioluminescence resonance energy transfer (BRET) to confirm the identity of the putative receptor. Finally, we generated a specific antibody against the newly detected PDFR of *E. rowelli* and localized this receptor protein in the animal. The obtained data provide insights into the onychophoran PDF/PDFR network and contribute to a better understanding of its evolution in panarthropods and ecdysozoans.

## Materials and Methods

### Collection and Maintenance of Specimens

Specimens of *Euperipatoides rowelli* Reid, 1996 (63) were obtained from decaying logs and leaf litter in the Tallaganda State Forest (New South Wales, Australia; 35°30′31″S, 149°36′14.3″E, 934 m) in October 2016. They were collected under the permit number SPPR0008 issued by the Forestry Commission of New South Wales and exported under the permit number WT2012-8163 provided by the Department of Sustainability, Environment, Water, Population and Communities. The collected specimens were maintained in the laboratory as described previously (64).

### Transcriptome *de novo* Assembly and Identification of Pigment-Dispersing Factor Receptor Genes

Illumina short reads from two specimens (male and female) of *E. rowelli*, sequenced as part of the i5K initiative (65, 66), were obtained from the short read archive (SRA) of GenBank (ER9 female: accession number SRR1946792; ER10 male: accession number SRR1946791). Prior to the assembly step, sequencing adapters were trimmed using cutadapt v1.8.1 (67) (parameters: –max-n = 0 -u 10 -u-5 -U 10 -U-5) and short reads with more than five bases below a Phred quality score of 15 were removed using ConDeTri v2.2 (68) (-hq = 14 -lq = 10 -sc = 33 -frac = 0.95 -minlen = 85). Both data sets were then assembled *de novo* using the software IDBA-Tran v1.1.1 (69) (–mink 20 –maxk 85 –step 5 –max_isoforms 1 –min_contig 100) yielding 92,426 unique contigs for ER9 (mean contig length = 717, N50 = 851) and 59,131 for ER10 (mean contig length = 707, N50 = 817), respectively. Both assemblies are available upon request.

To obtain the sequences of putative *pdfr* genes from the transcriptome of *E. rowelli*, two bait sequences of known pigment-dispersing factor receptors from the kuruma shrimp *Marsupenaeus japonicas* (accession number BAH85843.1) and the fruit fly *D. melanogaster* (NP_570007.2) were used in tBLASTn v2.4.0+ searches (70) against three assemblies in total [ER9 and ER10 from this study; Filter15 assembly from Hering et al. (62)] yielding 27 candidate sequences with an E value less than 1e^−5^ as a threshold.

### Cluster Analysis and Phylogenetic Reconstruction

After screening for putative pigment-dispersing factor receptor genes in *E. rowelli* transcriptomes, a cluster analysis of the 27 candidate sequences together with 18,337 bilaterian G protein-coupled receptors (GPCRs) obtained from the GPCR database (71) was performed using CLANS (72). In short, all sequences were clustered based on pairwise sequence similarities obtained from all-vs.-all BLAST searches. Because the best BLAST hit does not necessarily imply the closest relationship (73), a phylogenetic tree was reconstructed afterwards from all formerly obtained class B GPCRs, to which the PDFRs most likely belong (57, 59).

For phylogenetic analyses, the transmembrane domains of all class B GPCRs obtained from the cluster analysis [993 in total, mainly hormone receptors, including PDF receptors; reviewed in (74, 75)] were predicted using Pfam-A v29 (76, 77) and aligned using the MAFFT online version v7.452 (78) with the most accurate option L-INS-i and default parameters. To remove homoplastic and random-like positions, the alignment was masked with the software Noisy rel. 1.15.12 (79) (-seqtype = P -shuffles = 10,000) prior to the analyses. Two Maximum likelihood analyses were conducted with the Pthreads version of RAxML v8.2.10 (80). For each run, the best tree was obtained from a combined analysis (-f a option) of 10 independent inferences and GAMMA correction of the final tree under either the empirical JTT substitution model or a dataset-specific GTR substitution matrix, including calculation of bootstrap support values from 100 pseudoreplicates using the rapid bootstrapping algorithm implemented in RAxML. The JTT model was automatically selected by RAxML (PROTGAMMAAUTO option) as best-fitting substitution model. Notably, the analysis using the dataset-specific GTR+G model yielded a better log likelihood score (−87,233.49) for the best tree than using the best obtained empirical model JTT+G (−88,041.12). The phylogenetic tree was visualized with iTol v2 (81) and edited with Adobe Illustrator CS 5.1 (Adobe Systems Incorporated, San Jose, CA, USA).

### Amplification of Gene Fragments and Directional Cloning

The whole coding sequence (CDS) of the identified *Er-pdfr* gene was amplified from previously synthesized cDNA of *E. rowelli* [see (82)] using gene-specific primers (Er_pdfr_HindIII_F: 5′-tcgaagcttgccaccATGTGGTATTTAATTTATTGTATTTTATCCTTC-3′; Er_pdfr_XhoI_R: 5′-tgcgctcgagCTAAATACACAATTCCTCCAACAC-3′), which contained restriction sites (HindIII, XhoI) required for subsequent directional cloning into the mammalian expression vector pcDNA3.1(+) (Invitrogen, Carlsbad, CA, USA) to generate the plasmid pcDNA3.1-ErPDFR-3K10. The plasmid DNA was purified from 100 mL of transformed *E. coli* bacterial cell culture using the PureYield™ Plasmid Midiprep System (Promega GmbH, Walldorf, Germany) including an endotoxin removal step. The sequence and orientation of the cloned insert was verified by Sanger sequencing (Eurofins Genomics GmbH, Ebersberg, Germany) and deposited in GenBank under the accession number Er-pdfr MT080366.

### Antibody Generation

Polyclonal antibodies against Er-PDF-I and Er-PDF-II were newly generated by using HPLC-purified synthetic Er-PDF-I (CNAELINSLLGLPKMMNDA-NH2) and Er-PDF-II peptides (CNAELINSLLNLPQKLQEA-NH2; peptides&elephants GmbH (Hennigsdorf, Germany). Prior to immunization in rabbits, the peptides were coupled via an additional cysteine at the N-terminus to Sulfo-SMCC-activated bovine thyroglobulin as carrier. The anti-Er-PDF antibodies (anti-Er-PDF-I: IG-P1035, LOT# 2078B, 14 μg/mL; anti-Er-PDF-II: IG-P1036, LOT# 2079B2, 21 μg/mL) were purified from sera of immunized rabbits by two-fold depletion of one antibody against the antigen of the other and subsequent affinity purification on its own antigen to minimize cross-reactivity (ImmunoGlobe GmbH, Himmelstadt, Germany).

A HPLC-purified synthetic peptide from the C-terminal region of Er-PDFR (Ac-LRDHQGGQLSEDRRDC-NH2, Schafer-N ApS, Copenhagen, Denmark) was coupled to keyhole limpet hemocyanin (KLH) and used to newly generate a polyclonal antibody (anti-Er-PDFR; IG-P1044, LOT# 2130-5.6, 25 μg/mL), which was affinity purified from blood serum of an immunized goat (ImmunoGlobe GmbH).

### BRET Assay

HEK293T cells were seeded on 96-well Nunc plates (Thermo Fisher Scientific, Waltham, MA, USA) at a density of 2 ×10^4^ cells/well and cultured in DMEM high glucose (Sigma-Aldrich Chemie GmbH, Munich, Germany) supplemented with 10% fetal bovine serum and 5% CO_2_ at 37°C. After 24 h, cells were transfected using polyethyleneimine (25 kDa PEI, linear; Polysciences Europe GmbH, Hirschberg an der Bergstrasse, Germany) with GFP2-hEpac1E157-P881-Rluc8 [Epac-L; modified from (83–85)] and pcDNA3.1-ErPDFR-3K10 in a 1:1 ratio (100 ng total DNA/well). Cells were washed with HBSS (Thermo Fisher Scientific) 2 days after transfection and stimulated with different concentrations of synthetic PDF peptides (10^−11^ to 10^−5^ M final concentration) together with the substrate coelenterazine 400A (“DeepBlueC”; BIOTREND Chemikalien GmbH, Cologne, Germany) at a final concentration of 5 μM in a total volume of 50 μL HBSS. Control experiments were conducted with cells expressing Epac-L alone, stimulation without PDF peptides, stimulation using an unrelated peptide (myoinhibitory peptide 7 from the cockroach *Rhyparobia maderae*), and stimulation with forskolin (50 μM final concentration; Sigma-Aldrich Chemie GmbH) + IBMX (100 μM final concentration; Sigma-Aldrich Chemie GmbH), respectively. The raw luminescence was measured immediately after stimulation at wavelengths of 410 ± 80 nm for the donor and 515 ± 30 nm for the acceptor in eight biological replicates for each tested PDF peptide concentration (*n* = 8) using a POLARstar Omega microplate reader (BMG Labtech, Cary, NC, USA). Their ratios [emission (410 nm)/emission (515 nm)] were box plotted as relative luminescence against the respective PDF concentration in dose response curves.

### Western Blots and Specificity Tests

Western blots for goat anti-Er-PDF-I and goat anti-Er-PDF-II were performed using synthetic Er-PDF-I and Er-PDF-II peptides. Western blots for goat anti-Er-PDFR were conducted using synthetic peptide coupled to bovine serum albumin (BSA) as well as tissue lysate from dissected heads. For lysate preparation, five specimens of *E. rowelli* were anesthetized in chloroform vapor and dissected in cold phosphate-buffered saline (PBS; 0.1 M, pH 7.4). Tissues of onychophoran heads were homogenized on ice in a solution containing 500 μL NP-40 lysis buffer (pH 8.0) and the protease inhibitor cOmplete™ Mini (Roche Diagnostics GmbH, Mannheim, Germany). Tissue was incubated in lysis buffer for 2 h at 4°C. Subsequently, the lysed tissue was centrifuged and the supernatant stored at −80°C.

Synthetic Er-PDF-I and Er-PDF-II peptides were diluted in 4x Laemmli sample buffer (pH 6.8), heated for 5 min at 95°C and applied to an acrylamide Tris-tricine gel (10%) for 30 min at 30 V and 120 min at 50 V and then transferred to a Roti®-pvdf 0.2 membrane (Carl Roth GmbH & Co. KG, Karlsruhe, Germany) via semi-dry blot for 120 min at 84 mA. The synthetic Er-PDFR peptides were diluted in 2x Laemmli sample buffer, heated for 5 min at 95°C) and, together with the proteins of the lysate (lysed 1:1 in 2x Laemmli sample buffer), separated using SDS-PAGE on a 10% gel for 15 min at 30 V and 40 min at 200 V and then transferred to a Porablot NCP nitrocellulose membrane (Macherey-Nagel GmbH & Co. KG, Düren, Germany) via semi-dry blot for 60 min at 150 mA. Membranes were blocked for 30 min using 4% powdered milk in PBS at room temperature and then incubated with either rabbit anti-Er-PDF-I and rabbit anti-Er-PDF-II (1 μg/mL in PBS each) or goat anti-Er-PDFR (1 μg/mL in PBS) overnight at 4°C. Following several washing steps with PBS, membranes were incubated with either goat anti-rabbit or donkey anti-goat antibodies, respectively, conjugated with alkaline phosphatase (1:500; dianova GmbH, Hamburg, Germany) and washed again in PBS. The signal was developed using a solution containing 175 μg/mL BCIP (5-bromo-4-chloro-3-indolyl phosphate; Thermo Fisher Scientific) in dimethylformamide and the reaction was stopped with PBS.

In order to further test the specificity of the generated antibodies, additional western blots were conducted as described above with the following changes: Er-PDF-I antibody was tested against the Er-PDF-II peptide and, vice versa, the Er-PDF-II antibody was tested against the Er-PDF-I peptide.

### Whole-Mount Preparation, Vibratome Sectioning, and Immunohistochemistry

For immunohistochemistry, specimens were anesthetized in chloroform vapor and fixed according to Stefanini et al. (86) in 4% paraformaldehyde (PFA) containing 7.5% picric acid in phosphate buffered saline (PBS) overnight. Specimens were washed in several rinses of PBS, cut in halves and embedded in 31.25% albumin from chicken egg (Sigma-Aldrich Chemie GmbH) and 4.17% gelatin from porcine skin (Type A; Sigma-Aldrich Chemie GmbH) in distilled water. Albumin-gelatin blocks were hardened for 4 h at 4°C and then fixed in 10% PFA overnight. After several rinses in PBS, the blocks were cut in 80–100 μm thick sections using a vibratome (MICROM 650 V; Microm International GmbH, part of Thermo Fisher Scientific, Walldorf, Germany). The sections used for anti PDFR-labeling were treated with a mixture of collagenase/dispase (Roche Diagnostics GmbH; 1 mg/mL each) and hyaluronidase (Sigma-Aldrich Chemie GmbH; 1 mg/mL) in PBS for 40 min at 37°C and subsequently washed in PBS. Sections were blocked either in 10% normal goat serum (NGS; Sigma-Aldrich Chemie GmbH) for anti-Er-PDF-I and anti-Er-PDF-II labeling or in 10% bovine albumin serum (BSA; Carl Roth GmbH & Co. KG) for anti-Er-PDFR labeling in PBS containing 1% Triton X-100 (PBS-Tx, Sigma-Aldrich Co) for 1.5 h, followed by an incubation with the primary antibodies, either (i) rabbit anti-Er-PDF-I, (ii) rabbit anti-Er-PDF-II or (iii) goat anti-Er-PDFR (0.2 μg/mL in PBS-Tx, 1% NGS) for 2 days (rabbit anti-Er-PDF-I and rabbit anti-Er-PDF-II) or 5 days (goat anti-Er-PDFR) at 4°C. After several changes of PBS for 8–24 h, sections were incubated with either goat anti-rabbit Alexa Fluor® 488 (1:500; Thermo Fisher Scientific) or donkey anti-goat Alexa Fluor® 488 or Alexa Fluor® 680 (1:500; Thermo Fisher Scientific) for 2 days at 4°C. After washing steps in PBS-Tx, sections were further washed using PBS alone and then incubated in a solution containing 4′,6-diamidino-2-phenylindole (DAPI; 1 ng/mL; Carl Roth GmbH & Co. KG) and phalloidin-rhodamine (25 μg/mL in PBS; Thermo Fisher Scientific) for 2 h at room temperature. After rinsing the sections several times in PBS they were mounted between two coverslips in ProLong™ Gold Antifade Reagent (Invitrogen).

For double labeling of both PDF peptides, the procedure was the same as for single labeling with the following changes: the sections were incubated in 10% BSA in PBS-Tx for 1.5 h. Thereafter, the sections were first incubated with the rabbit anti-Er-PDF-II (0.2 μg/mL in PBS-Tx, 1% BSA) at 4°C for 2 days. After several changes of PBS-Tx for 12 h, the sections were then incubated in a solution containing the fab fragments goat anti-rabbit (Jackson Immuno Research Europe Ltd., Cambridgeshire, UK; 40 μg/mL in PBS-Tx, 1% BSA) for 2 days at 4°C. After changes of PBS-Tx for 8–24 h, the sections were incubated in donkey anti-goat Alexa Fluor® 594 (1:500, Thermo Fischer Scientific). Thereafter, the sections were incubated with rabbit anti-Er-PDF-I (0.2 μg/mL in PBS-Tx, 1% BSA) at 4°C for 2 days, followed by an incubation in goat anti-rabbit Alexa Fluor®488 (1:500; Thermo Fischer Scientific) and counterstained with DAPI (Carl Roth GmbH & Co. KG).

For double labeling with anti-Er-PDFR and either rabbit anti-Er-PDF-I or rabbit anti-Er-PDF-II, the procedure was as described for single labeling with the following changes: the incubations with the primary antibodies anti-Er-PDFR with either rabbit anti-Er-PDF-I or rabbit anti-Er-PDF-II (0.2 μg/mL in PBS-Tx) as well as the secondary antibodies donkey anti-goat Alexa Fluor® 488 and donkey anti-rabbit® 568 (1:500; Thermo Fisher Scientific) were performed simultaneously for 5 days at 4°C.

Additional double labeling of both PDF peptides was performed on whole mount-preparations of the brain. Specimens of *E. rowelli* were first anesthetized in chloroform vapor and their brains were dissected in physiological saline (87). Following the fixation after Stefanini et al. (86) for 3 h at room temperature, the brains were washed in PBS, dehydrated through an ascending ethanol series (70, 90, 95, 100, and 100%, 10 min each), cleared in xylene for 10 min twice, rehydrated through a descending ethanol series (100, 100, 95, 90, 70 and 50%, 5 min each) and washed several times in PBS. The samples were then treated with a mixture of collagenase/dispase (Roche Diagnostics GmbH; 1 mg/mL each) and hyaluronidase (Sigma-Aldrich Chemie GmbH; 1 mg/mL) in PBS for 40 min at 37°C and washed in several rinses of PBS. The staining procedure was as described for sections with the following modifications: the incubation times of the primary antibodies, the fab fragments and the secondary antibodies were increased to 4 days. After the incubation with the secondary antibodies, the samples were washed in PBS-Tx, dehydrated through an ascending ethanol series (70, 90, 95, 100, and 100%, 10 min each), cleared and mounted between two coverslips in methyl salicylate using custom-made carbon fiber-reinforced polymer microscope slides as spacers.

### Confocal Laser Scanning Microscopy and Image Processing

Whole mount preparations and vibratome sections were analyzed using a confocal laser scanning microscope (Zeiss LSM 880; Carl Zeiss Microscopy GmbH, Jena, Germany) equipped with an Airyscan module. Images were acquired and raw Airyscan datasets processed using the ZEN 2 (Black edition) imaging software (Carl Zeiss Microscopy GmbH). Selected substacks were created and adjusted to optimal brightness and contrast using Fiji v.1.52 (88, 89). Panels and diagrams were designed using Illustrator CS5.1 and the video was processed with Adobe Premiere Pro CS5.1 (Adobe Systems Incorporated).

## Results

### Identification of the Onychophoran PDF Receptor

**Figure 2:**
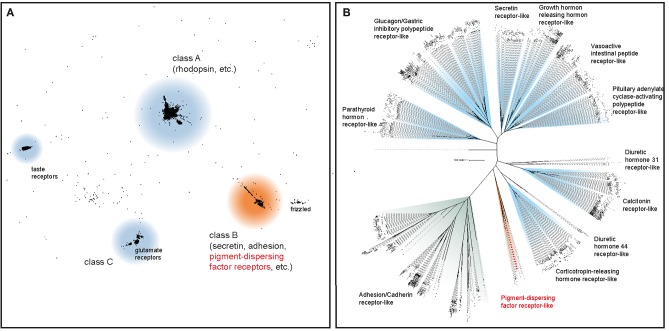
Phylogenetic analyses to deorphanize the PDF receptors of *E. rowelli*. **(A)** Cluster analysis of ~18,300 bilaterian G protein-coupled receptor genes (GPCRs) including 27 PDF receptor candidate sequences from *E. rowelli*. The position of the PDF receptor family is highlighted in red. **(B)** Maximum likelihood tree under a dataset-specific GTR+G model of amino acid sequence evolution of ~1,000 class B GPCRs (including PDF receptors) obtained from the former cluster analysis. Note the occurrence of a putative PDF receptor from *E. rowelli* in a clade of protostome PDF receptors (highlighted in red). See Supplementary Figures 1, 2 for the full trees.

The initial BLAST search for PDF receptor homologs in three transcriptomes of *E. rowelli* yielded 27 candidate sequences. We included these in subsequent phylogenetic analyses of known bilaterian GPCRs to obtain the onychophoran PDFR ortholog. After prescreening the most likely candidate sequences in an all-vs.-all BLAST cluster analysis (Figure 2A), a maximum likelihood tree was reconstructed using the formerly obtained class B GPCR members, to which PDF receptors most likely belong (57, 59). Using this approach, we identified one PDF receptor gene (*Er-pdfr*) in each analyzed transcriptome of *E. rowelli*; only in one of the transcriptomes (ER10) the respective transcript was fractured in two contigs (transcripts 10,426 and 39,667; see Supplementary Figures 1, 2). The detected *Er-pdfr* sequence clearly falls into the monophyletic group of protostome PDFRs (Figure 2B; Supplementary Figures 1, 2).

### *In vitro* Analysis of the Functionality of the Identified PDF Receptor

**Figure 3:**
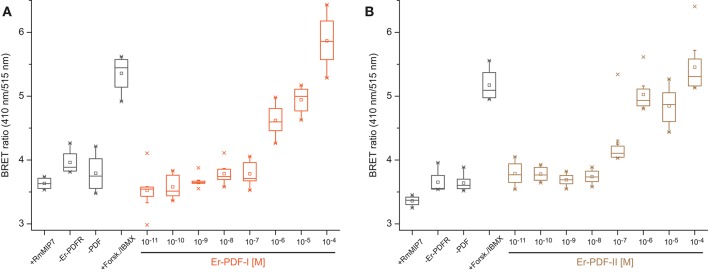
Dose-response curves depicting BRET ratio changes of HEK293T cells transfected with the PDF receptor Er-PDFR of *E. rowelli* together with the Epac-L cAMP-sensor after exposure to different concentrations of the synthetic PDF peptides Er-PDF-I **(A)** and Er-PDF-II **(B)**. Experiments without transfecting Er-PDFR (–ErPDFr), without Er-PDF stimulus (–PDF) and stimulating with an unrelated peptide (myoinhibitory peptide; +RmMIP7) were conducted as negative controls. Forskolin/IBMX (+Forsk./IBMX), stimulating adenylyl cyclase and inhibiting phosphodiesterases, respectively, was induced as a positive control in order to generate a massive increase of cAMP within the cell [reviewed in Prinz et al. (90)]. Box plots of eight biological replicates each (*n* = 8). Interquartile range (IQR) is delimited by the 25th (lower border) and 75th percentile (upper border); whiskers depict the 1.5-fold IQR. The median (50th percentile) is indicated by a horizontal line within the box, while squares (□) demarcate the mean, and crosses ( × ) the minimum or maximum values, respectively.

To test the functionality of the recognized Er-PDF receptor, we further performed bioluminescence resonance energy transfer (BRET) experiments on cultured human cells (HEK293T) using an Epac-based cAMP-sensor (Epac-L). The cAMP response data revealed that the transfected Er-PDFR is activated by both PDF peptides of *E. rowelli*, Er-PDF-I and Er-PDF-II, in a dose-dependent manner (Figures 3A,B). In contrast, the myoinhibitory peptide Rm-MIP7, which is co-localized with PDF in circadian pacemaker cells of the cockroach *Rhyparobia maderae* [see (91)] and which we used as a control, did not stimulate the receptor of *E. rowelli*. The minimum concentrations required for PDFR stimulation differs between the two PDFs: while Er-PDF-I showed an activation threshold at 10^−6^ M, the receptor sensitivity to Er-PDF-II was lower by one order of magnitude at 10^−7^ M (Figures 3A,B).

### Specificity Tests of Er-PDF-I, Er-PDF-II, and Er-PDFR Antibodies

**Figure 4:**
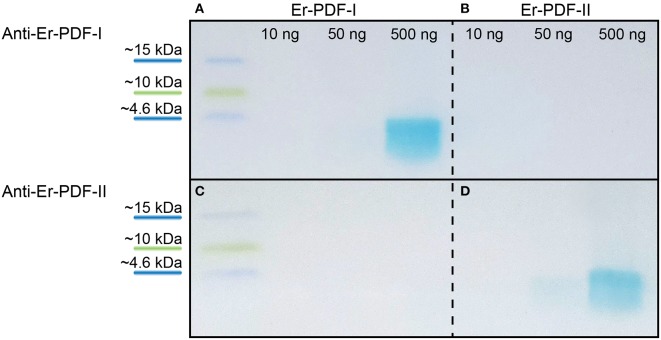
Western blot analyses testing specificity and putative cross-reactivity of anti-Er-PDF-I and anti-Er-PDF-II of *E. rowelli*. The loaded synthetic peptide is indicated on the top. Anti-Er-PDF-I recognizes the synthetic Er-PDF-I peptide at a concentration of 500 ng **(A)** but shows no reactivity with the synthetic Er PDF-II peptide **(B)**. While synthetic Er-PDF-II is recognized by anti-Er-PDF-II using 50 and 500 ng synthetic peptide **(D)**, synthetic Er-PDF-I is not recognized by anti-Er-PDF-II **(C)**.

To test the specificity of the generated Er-PDF-I and Er-PDF-II antibodies, we performed western blot analyses using different concentrations of synthetic peptides (Figures 4A–D). The western blots show a distinct band below 4.6 kDa for the Er-PDF-I antibody (tested on 500 ng of synthetic Er-PDF-I peptide) and the Er-PDF-II antibody (using 50 ng and 500 ng of synthetic Er-PDF-II peptide), respectively (Figures 4A,D). The control experiments reciprocally using the non-corresponding peptides revealed no signal (Figures 4B,C).

**Figure 5:**
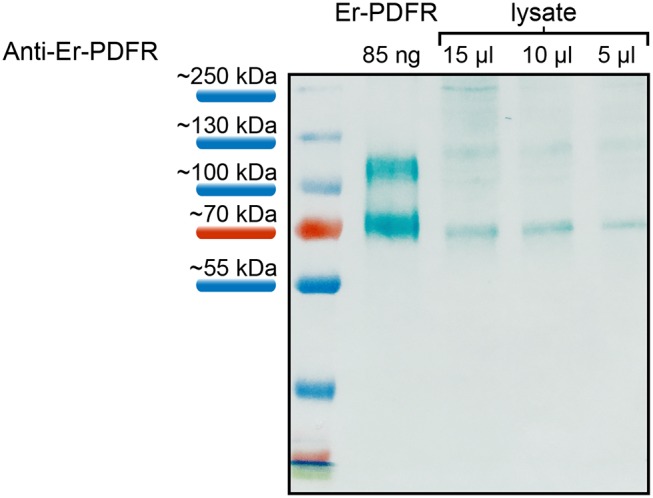
Western blot analyses of the specificity of anti-Er-PDFR using a synthetic truncated Er-PDFR peptide-BSA fusion (first lane) and tissue lysates from heads of *E. rowelli* at decreasing concentrations (second to last lanes). The Er-PDFR antibody recognized the synthetic Er-PDFR peptide-BSA fusion (expected molecular weight of 61 kDa) at ~70 kDa and PDFR within all loaded lysate concentrations. Note that the second band in all lanes might indicate dimerization of the synthetic Er-PDFR peptide-BSA fusion (first lane) and Er-PDFR of the head lysate lanes, which are recognized by the Er-PDFR antibody.

The specificity of the Er-PDFR antibody was tested using both the synthetic peptide as well as lysates of entire heads of *E. rowelli* at different dilutions (Figure 5). The western blot employing the synthetic PDFR peptide coupled to BSA exhibits a distinct band at ~70 kDa and an additional band above 100 kDa. The western blots using different dilutions of lysates show distinct bands at ~70 kDa and a considerably weaker band at ~120 kDa in all three lanes (Figure 5). The lane carrying 15 μl of lysate exhibits an additional weak band at ~250 kDa.

### Immunolocalization of PDF-I and PDF-II Peptides and Their Receptor

**Figure 6:**
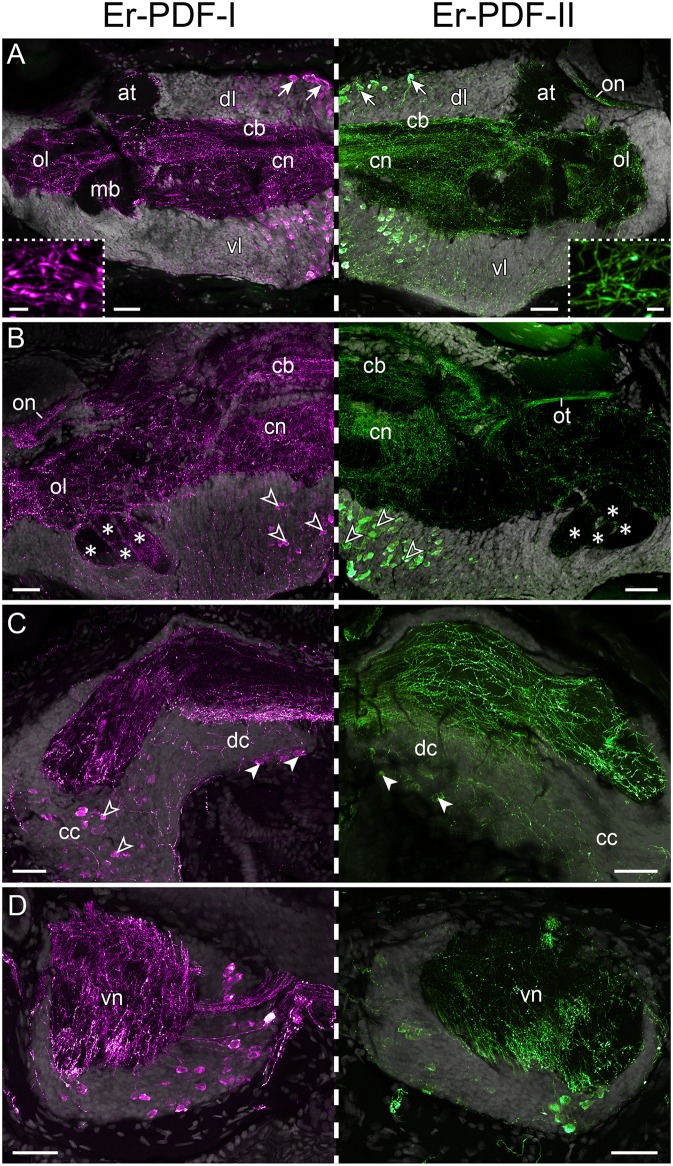
Immunolocalization of Er-PDF-I and Er-PDF-II in *E. rowelli*. Confocal laser scanning micrographs of vibratome sections. Dorsal is up in all images. Hatched line indicates median regions. Er-PDF-I-ir (magenta), Er-PDF-II-ir (green) and DNA-labeling (gray) from anterior to posterior through head **(A–C)** and trunk **(D)**. Note similar distribution of either peptide in brain and ventral nerve cords. **(A)** Arrows indicate dorsal groups of somata in protocerebrum. Insets show large varicosities in PDF-immunoreactive fibers. **(B)** Arrowheads point to large ventral groups of somata in protocerebrum. Asterisks indicate four lobes of mushroom bodies. **(C)** Filled arrowheads point to somata in deutocerebrum. Empty arrowheads demarcate somata in connecting cords. **(D)** Cross sections of ventral nerve cords. at, antennal tract; cb, central body; cc, connecting cord; cn, central neuropil; dc, deutocerebrum; dl, dorsal perikaryal layer; ol, olfactory lobe; on, optic neuropil; ot, optic tract; vl, ventral perikaryal layer; vn, neuropil of ventral nerve cord. Scale bars: 50 μm **(A–D)** and 500 nm (insets).

**Figure 7:**
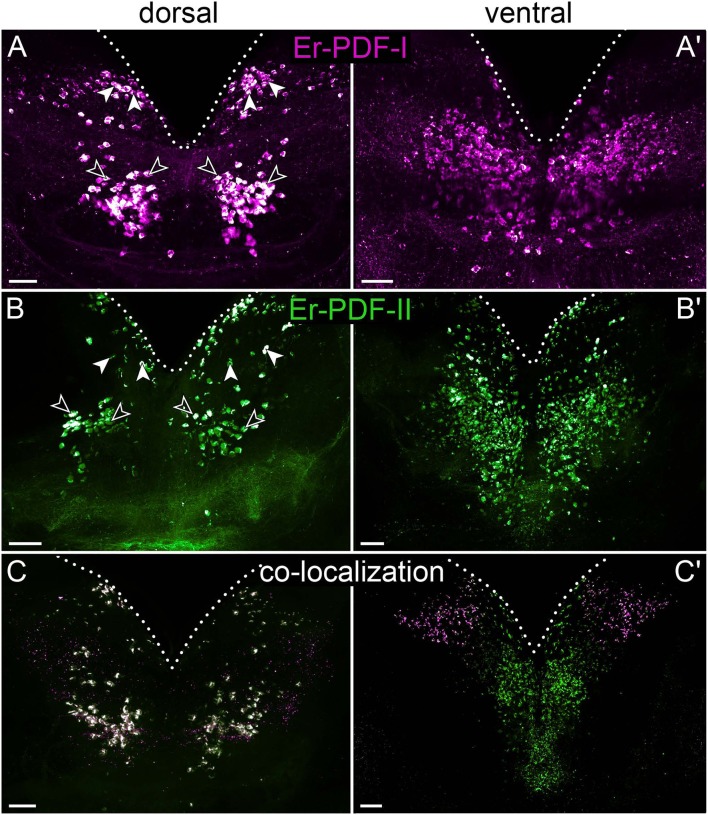
Combined immunolocalization of Er-PDF-I and Er-PDF-II in the brain of *E. rowelli*. Maximum projection of a substack of confocal laser scanning micrographs. Dotted lines indicate outline of brain. Anterior is up in all images. Er-PDF-I-ir (magenta), Er-PDF-II-ir (green), and co-localization (white). **(A,B)** Dorsal Er-PDF-I-ir /Er-PDF-II-ir cell group is subdivided in anterior (filled arrowheads) and median cell groups (open arrowheads). Ventrally located somata with strong Er-PDF-I immunoreactivity occur anteriorly and laterally **(A′,C′)**, whereas those with strong Er-PDF-II immunoreactivity are located further posteriorly **(B′,C′)**. **(C)** Note that all Er-PDF-I-ir and Er-PDF-II-ir somata of dorsal group appear in white, indicating co-localization of both peptides at similar levels. Scale bar: 50 μm.

Immunolabeling against the Er-PDF-I and Er-PDF-II peptides revealed high numbers of somata and fibers in the brain and the ventral nerve cords of *E. rowelli* (Figures 6A–D, 7A–C,A′–C′). In each brain hemisphere, Er-PDF-I and Er-PDF-II immunoreactive (ir) somata occur mainly in one large ventromedian (Figures 7A′–C′) and two dorsal groups, the latter comprising a smaller anterior and a larger median group (Figures 7A–C). While the somata of the anterior group innervate the anterior neuropil, the larger median group is associated with the central body and the central neuropil and is located closer to the dorsal surface of the brain (Figures 6A,B; Supplementary Video 1). The somata of the ventromedian group occupy mainly the median portion of the ventral perikaryal layer, in which they are situated at different depths (Figures 6A,B, 7A′,B′). Notably, no Er-PDF-I-ir or Er-PDF-II-ir somata are found in the so-called hypocerebral organs–vesicle-like structures associated with the ventral cortex of each brain hemisphere (Supplementary Figures 3A,B). Additional groups of Er-PDF-I-ir and Er-PDF-II-ir somata occur in the ventral part of the deutocerebrum as well as in the connecting cords, which link the brain with the ventral nerve cords (Figure 6C). Within the ventral nerve cords, the somata are mainly located in the ventral and median perikaryal layers (Figure 6D). While most somata with a strong Er-PDF-II signal appear ventrally, those with a strong Er-PDF-I signal show a wide mediolateral distribution in each nerve cord.

In addition to somata, our immunolabeling revealed numerous Er-PDF-I-ir and Er-PDF-II-ir fibers, which are characterized by typical varicosities and occur in all major neuropils of the *E. rowelli* brain including the central neuropil, the central body, the mushroom bodies, the olfactory lobes, and the optic tracts and neuropils (Figures 6A–C). Despite the nearly ubiquitous distribution of Er-PDF-I-ir/Er-PDF-II-ir fibers in all brain neuropils, their density differs among specific regions. While the central neuropil shows a dense mesh of internal Er-PDF-I-ir and Er-PDF-II-ir fibers, the olfactory glomeruli are rather surrounded by them (Figures 6A,B). The lowest density of Er-PDF-I-ir and Er-PDF-II-ir fibers is found in the antennal tracts and the mushroom bodies, both of which exhibit fibers mainly in the periphery, except for the median lobe of the mushroom body, which additionally shows strong internal Er-PDF-I immunoreactivity (Figure 6B). In contrast, the hypocerebral organs do not contain any Er-PDF-I-ir or Er-PDF-II-ir fibers (Supplementary Figures 3A,B). Like in the brain, numerous Er-PDF-I-ir and Er-PDF-II-ir fibers are evident in the neuropils of each nerve cord (Figure 6D). Most fibers with a prominent Er-PDF-II immunoreactivity are confined to the ventral portion of the ventral cord neuropil, whereas those exhibiting strong Er-PDF-I signal are more widely distributed, thus reflecting the wide distribution of the corresponding somata in the perikaryal layer of the nerve cord.

**Figure 8:**
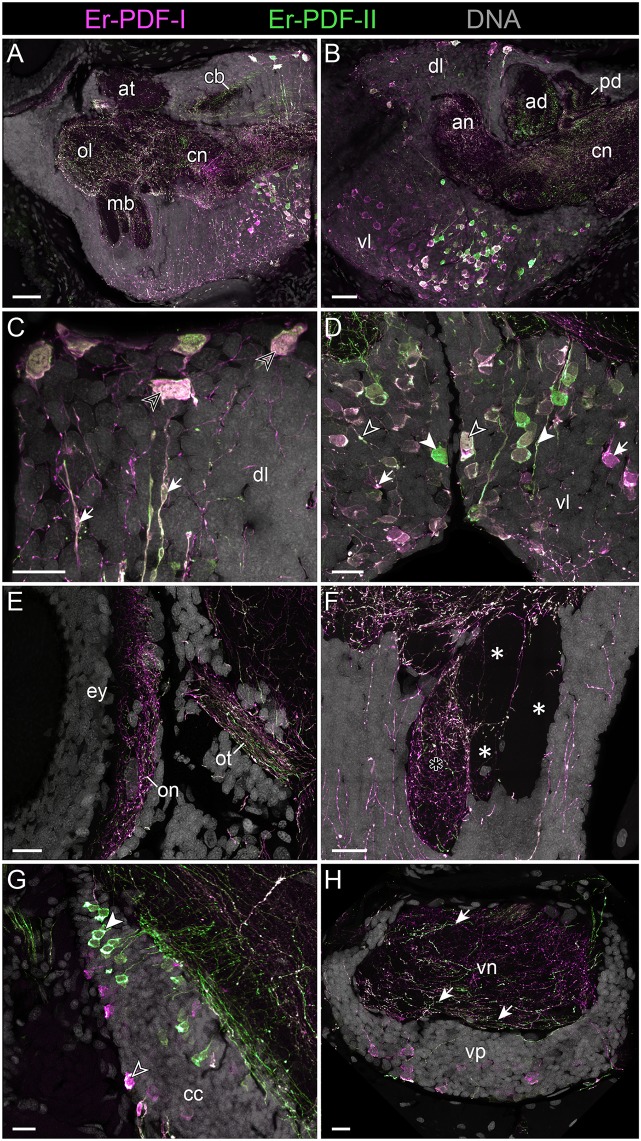
Combined immunolocalization of Er-PDF-I and Er-PDF-I in the brain of *E. rowelli*. Confocal laser scanning micrographs of vibratome sections. Dorsal is up in all images and anterior is left in **(B,G)**. Er-PDF-I-ir (magenta), Er-PDF-II-ir (green) and DNA (gray). **(A,B)** Er-PDF-I and Er-PDF-II are co-localized in neuropil and somata, albeit at different intensities. **(C)** Somata (arrowheads) and processes (arrows) of dorsal cell group exhibit equal intensity levels of Er-PDF-I-ir and Er-PDF-II-ir. **(D)** In contrast, somata and processes of ventral cell group occur in three variants: (i) Er-PDF-I-ir and Er-PDF-II-ir at equal levels (open arrowheads), (ii) Er-PDF-I-ir at higher level (arrows), and (iii) Er-PDF-II-ir at higher level (filled arrowheads). **(E–H)** Differences in expression levels of Er-PDF-I-ir and Er-PDF-I-ir are also seen in optic neuropil **(E)**, inner lobe (black asterisk) as opposed to remaining lobes of the mushroom bodies (white asterisks) **(F)**, somata of connecting cords **(G)**, and somata and neuropil of nerve cords **(H)**. an, anterior neuropil; ad, anterior division of central body; at, antennal tract; cb, central body; cc, connecting cord; cn, central neuropil; dl, dorsal perikaryal layer; ey, eye; mb, mushroom body; ol, olfactory lobe; on, optic neuropil; ot, optic tract; pd, posterior division of the central body; vl, ventral perikaryal layer; vn, neuropil of ventral nerve cord; vp, perikaryal layer of ventral nerve cord. Scale bars: 50 μm **(A,B)** and 20 μm **(C–H)**.

To determine whether Er-PDF-I and Er-PDF-II are co-expressed at least in some cells and regions of the nervous system, we performed double labeling. Our data indeed show that both peptides are found in the same sets of neuronal somata and fibers within the brain and the ventral nerve cords (Figures 7C, 8A–H). However, the signal intensity differs markedly between tissues and cells. For example, while Er-PDF-I and Er-PDF-II are both expressed at a high level in the dorsal groups of somata within the brain (Figures 7C, 8A–C), the ventromedian groups show a more sophisticated pattern, with Er-PDF-I exhibiting a stronger signal in the anterolateral and Er-PDF-II in the median regions (Figures 7C′, 8A,B,D). A differentiated signal is evident even within individual cells. While some somata show a completely overlapping signal, others seem to express either Er-PDF-I or Er-PDF-II (Figures 7C,C′, 8A–D). However, high resolution imaging confirmed the presence of both peptides in all labeled somata, although they seem to be expressed at different levels (Supplementary Figures 4, 5).

**Figure 9:**
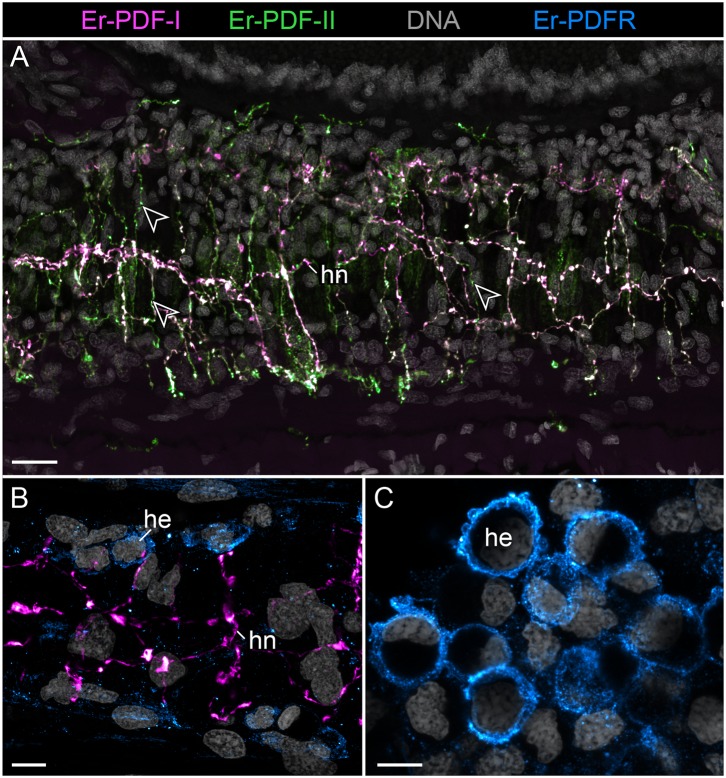
Combined immunolocalization of either Er-PDF-I or Er-PDF-I with Er-PDFR in the vascular system of *E. rowelli*. Confocal laser scanning micrographs of vibratome sections. Anterior is left in all images. Er-PDF-I (magenta), Er-PDF-II (green), Er-PDFR (cyan), and DNA (gray). **(A)** Er-PDF-I and Er-PDF-II are co-localized in heart nerve with varying intensities, especially within fine branches (arrowheads). **(B,C)** PDFR immunoreactivity in cell membranes of hemocytes found in heart lumen **(B)** and body cavity **(C)**. he, hemocyte; hn, heart nerve. Scale bars: 25 μm **(A)**, 10 μm **(B)**, and 5 μm **(C)**.

Different signal intensities are also evident among the fibers. Like the somata, the fibers show three different expression patterns with either both peptides expressed similarly or one or the other occurring at a higher level (Figures 8A–H, 9A). Differences are evident, for example, in the central body, which shows a stronger Er-PDF-II signal (Figure 8B), whereas the optic neuropil exhibits a higher expression of Er-PDF-I (Figure 8E). Double labeling of the mushroom body confirms the predominant occurrence of Er-PDF-I inside the median lobe (Figure 8F), which was detected by separately labeling the individual peptides (cf. Figure 6B). Likewise, the double labeling confirms the differences in the distribution of Er-PDF-I and Er-PDF-II in the neuropil of the ventral nerve cords, which shows a condensation of fibers with a strong Er-PDF-II signal in its ventral portion but a more ubiquitous distribution of fibers with a strong Er-PDF-I signal (Figure 8H).

Although the somata of the Er-PDF-I-ir and Er-PDF-II-ir neurons are exclusively found in the central nervous system of *E. rowelli*, it is worth mentioning that at least some of their fibers extend into the peripheral nervous system including various peripheral nerves (such as leg nerves, slime papilla nerves, tongue nerve, oral and pharyngeal nerves), the ring commissures, and the heart nerve (Figure 9A). In the heart wall, the fibers show the same differential expression pattern of the two peptides as in the remaining nervous system. While the fibers of the longitudinal, dorsomedian heart nerve express both peptides at a similar level, the associated transverse fibers exhibit a higher intensity of Er-PDF-II signal than Er-PDF-I. Remarkably, the high resolution confocal microscopy used for imaging reveals diverging patterns of Er-PDF-I and Er-PDF-II immunoreactivity even within the individual fibers in our specimens (Figures 8C,D, 9A).

**Figure 10:**
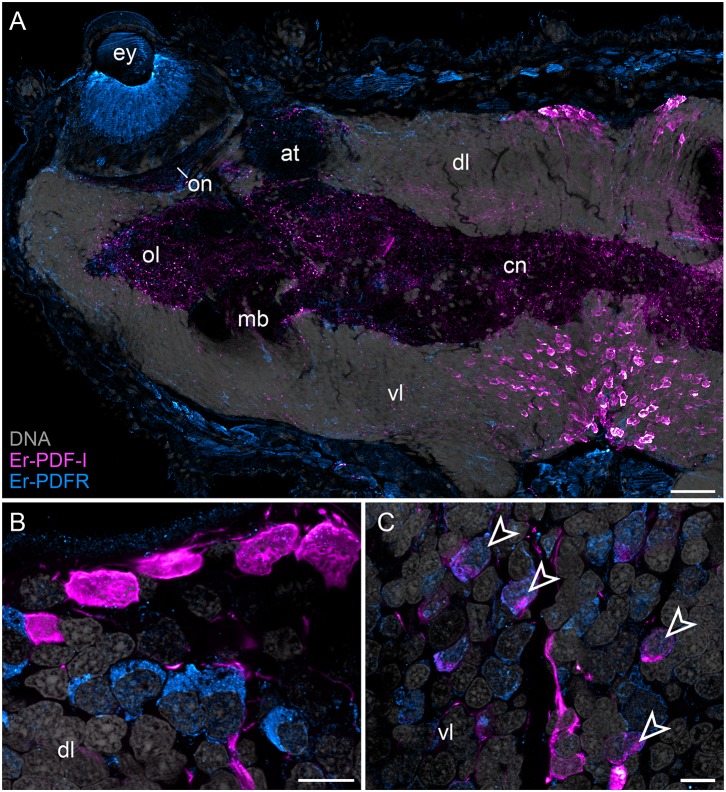
Combined immunolocalization of Er-PDF-I and Er-PDFR in *E. rowelli*. Confocal laser scanning micrographs of vibratome sections. Dorsal is up in all images. Er-PDF-I (magenta), Er-PDFR (cyan), and DNA (gray). Note that cuticle is autofluorescent. **(A)** Overview of protocerebrum. **(B)** Detailed view of dorsal perikaryal layer. **(C)** Detailed view of ventral perikaryal layer. Note that Er-PDF-I and Er-PDFR are co-localized only in some cells of ventral group (arrowheads). at, antennal tract; cn, central neuropil; dl, dorsal perikaryal layer; ey, eye; mb, mushroom body; ol, olfactory lobe; on, optic neuropil; vl, ventral perikaryal layer; Scale bars: 50 μm **(A)** and 10 μm **(B,C)**.

**Figure 11:**
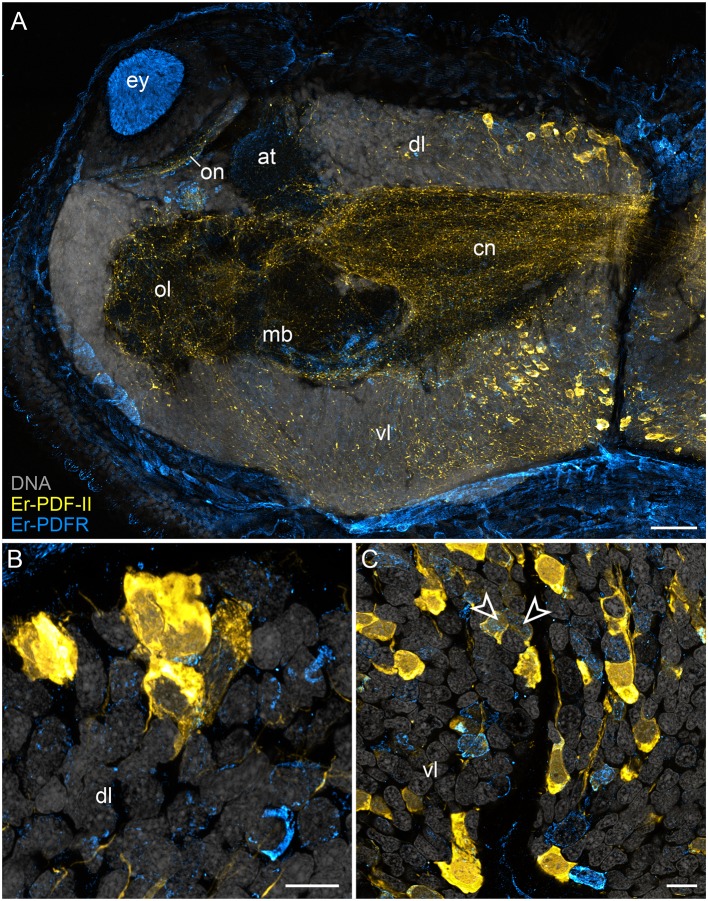
Combined immunolocalization of Er-PDF-II and Er-PDFR in *E. rowelli*. Confocal laser scanning micrographs of vibratome sections. Dorsal is up in all images. Er-PDF-II (yellow), Er-PDFR (cyan), and DNA (gray). Note that cuticle is autofluorescent. **(A)** Overview of protocerebrum. **(B)** Detailed view of dorsal perikaryal layer. **(C)** Detailed view of ventral perikaryal layer. Note that Er-PDF-II and Er-PDFR are co-localized in some cells of the ventral group (arrowheads). at, antennal tract; cn, central neuropil; dl, dorsal perikaryal layer; ey, eye; mb, mushroom body; ol, olfactory lobe; on, optic neuropil; vl, ventral perikaryal layer; Scale bars: 50 μm **(A)** and 10 μm **(B,C)**.

In addition to the Er-PDF-I and Er-PDF-II peptides, we immunolocalized the newly identified and deorphanized pigment-dispersing factor receptor (PDFR) of *E. rowelli*. As expected from its nature as a membrane receptor protein, the Er-PDFR immunoreactivity is generally found in the periphery of each cell (e.g., Figures 9B,C). Like the two analyzed PDF peptides, Er-PDFR is localized in a dorsal and a ventral group of somata within the brain (Figures 10A–C, 11A–C). However, double labeling with either Er-PDF-I or Er-PDF-II revealed no co-expression of Er-PDFR in the dorsal groups of neuronal somata, although at least some PDFR-ir cells are either directly adjacent to or located in close proximity to the Er-PDF-I-ir/Er-PDF-II-ir somata (Figures 10B, 11B). In contrast to the dorsal group, the Er-PDFR-ir somata of the ventral group show three different conditions: they are either (i) not closely associated with the Er-PDF-I-ir/Er-PDF-II-ir somata, (ii) directly adjacent to them, or (iii) co-express the two peptides and the receptor (Figures 10C, 11C). The hypocerebral organs exhibit no PDFR-ir somata (Supplementary Figure 3C).

**Figure 12:**
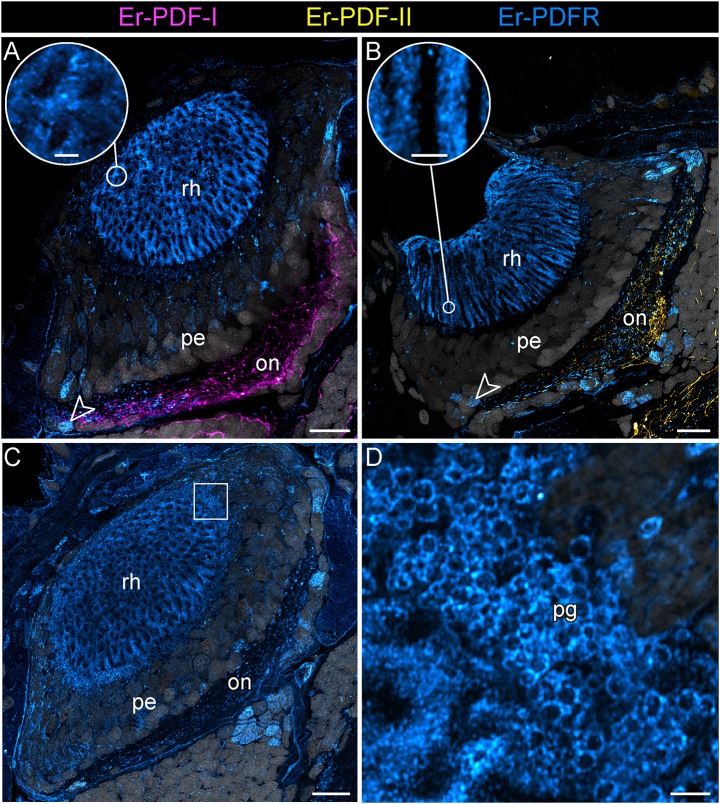
Combined immunolocalization of either Er-PDF-I or Er-PDF-I with Er-PDFR in the visual system *E. rowelli*. Confocal laser scanning micrographs of vibratome sections. Dorsal is up in all images. Er-PDF-I (magenta), Er-PDF-II (yellow), Er-PDFR (cyan) and DNA (gray). **(A–D)** PDFR immunoreactivity occurs in optic neuropil, few somata of optic ganglion (arrowheads), rhabdomeric layer (**A–C** and insets for detail), and pigment granules **(C,D)**. Square in **(C)** indicates region of **(D)**. Note the lack of co-localization od Er-PDFR with Er-PDF-I **(A)** or ER-PDF-II **(B)**. on, optic neuropil; pe, perikaryal layer of eye; pg, pigment granules rh, rhabdomeric layer; layer. Scale bars: 20 μm **(A–C)** and 2 μm (insets of **A**, **B,D**).

Besides the dorsal and ventral perikaryal layers of the brain, additional Er-PDFR-ir somata occur, for example, next to the olfactory glomeruli (Supplementary Figure 6A) and the median lobe of the mushroom body (Supplementary Figure 6B). Moreover, Er-PDFR is expressed in a few dorsolateral somata of the brain near the eye as well as in some somata of the optic ganglion itself (Figures 10A, 11A, 12A–C). Er-PDFR-expressing somata are absent in the ventral nerve cords, but are abundant in the connecting cords (Supplementary Figures 6C,D). Here, double labeling solely revealed somata with either Er-PDFR or Er-PDF-I/Er-PDF-II signal, whereas co-expression of both the peptide and the receptor is not evident.

In addition to the described Er-PDFR immunoreactivity in the neuronal somata, the receptor is identifiable in the neuropils of the brain, the eyes and the ventral nerve cords (Figures 10A, 11A, Supplementary Figures 6A–C). However, the signal in the neuronal processes is generally weaker and less defined than in the somata. Single fibers are thus difficult to follow and appear as numerous dots in each neuropil region (Supplementary Figures 6A–C).

Notably, in contrast to the PDF signal, Er-PDFR immunoreactivity is not restricted to the nervous system of *E. rowelli* but is additionally found in other cells and tissues (Figures 9B,C, 10A, 11A, 12A–D). The most prominent signal outside the nervous system occurs in the visual system including the microvilli of the photoreceptor processes (Figures 12A,B) and the pigment granules [cf. 86)] of the supportive cells (Figures 12C,D). Er-PDFR immunoreactivity is further associated with membranes of certain blood cells (= hemocytes), which appear blue under the bright-field microscope (Figures 9B,C, Supplementary Figures 7A,B). These blueish Er-PDFR-ir hemocytes do not only occur in the heart lumen but also in other regions of the circulatory system of *E. rowelli* (Figure 9C).

## Discussion

### Deorphanization of the Onychophoran PDF Receptor

Using a combination of transcriptomic and phylogenetic analyses, we were able to identify a putative PDF receptor candidate in the onychophoran *E. rowelli*. PDFRs are generally involved in cAMP-mediated signaling pathways and—in response to PDF—increase intracellular cAMP levels *in vivo* (92) and also *in vitro*, for example, when expressed in human embryonic kidney cells [HEK293; (57)]. We used the latter approach for clarifying whether or not the identified receptor is functional and whether it responds to both peptides by measuring its activity in the presence of either Er-PDF-I or Er-PDF-II *in vitro*. This was done by detecting the cAMP-induced activation of an Epac-based sensor [“Exchange protein activated by cAMP”; (93)] using bioluminescence resonance energy transfer [BRET; (84, 90, 94)]. Our data revealed that Er-PDFR is activated by both peptides, although Er-PDF-II seems to be one order of magnitude more potent than Er-PDF-I. A similar result was obtained from nematodes (41), but the reason for the observed differences in stimulation of the PDF receptor by different ligands remains unknown. Taken together, the results of our phylogenetic analyses and BRET experiments suggest that the identified receptor is indeed functional and most likely represents the sole PDF receptor of the onychophoran *E. rowelli*. Our BRET data further suggest that Er-PDFR is activated by Er-PDF-I and Er-PDF-II in a distinct and dose-dependent manner.

### Specificity of the Generated Antibodies

The western blots conducted for testing the affinity of the newly generated antibodies against Er-PDF-I and Er-PDF-II revealed specific bands below 4.6 kDa, corresponding to the expected molecular weight of both peptides of 1.9–2.0 kDa each. Specificity tests using the two antibodies against each non-corresponding synthetic peptide (anti Er-PDF-I and anti Er-PDF-II) were negative, suggesting that the two antibodies are specific and do not cross-react.

Additional western blots for testing the affinity of the antibody generated against the PDF receptor of *E. rowelli* yielded more intricate but consistent results. For these tests, we used a synthetic PDFR peptide coupled to BSA together with a lysate of dissected heads at different dilutions. All of these tests revealed a distinct band at ~70 kDa corresponding well to the expected molecular weight of the synthetic PDFR peptide-BSA conjugate (which is ~6 kDa: 1.9 kDa fractionized Er-PDFR plus 66 kDa BSA) and the entire receptor protein (61.06 kDa) in the head lysate. The appearance of additional bands at ~120 kDa and ~250 kDa may have been induced by the formation of dimers and multimers by the receptor protein itself/the BSA coupled to the synthetic peptide. We believe that this result does not compromise the specificity of the Er-PDFR antibody generated for this study, as it might be due to a technical artifact.

### Differential Expression of PDF-I and PDF-II Peptides in the Onychophoran Nervous System

**Figure 13:**
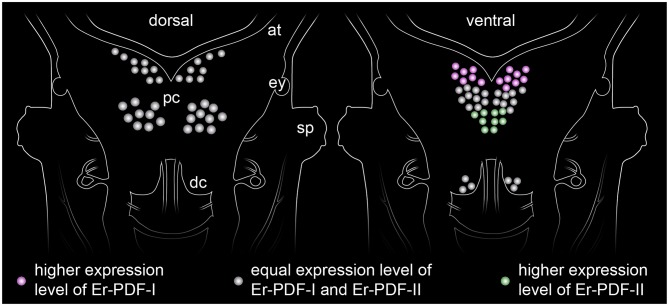
Simplified diagram illustrating the distribution of somata expressing the two PDF peptides (PDF-I and PDF-II) in the onychophoran brain. The somata of the dorsal cell group (diagram on the left) express both peptides at equal levels, whereas those of the ventral cell group show a more elaborate pattern of expression (diagram on the right). While the anterior somata exhibiting higher levels of PDF-I and the posterior ones of PDF-II, the median somata express both peptides at similar levels.

Our immunolabeling using two specific antibodies against the PDF-I and PDF-II peptides of *E. rowelli* largely confirms the broad distribution of PDFs in the onychophoran nervous system demonstrated previously (38). In accord with these previous findings, we encountered PDF immunoreactivity in groups of neuronal somata in the ventral and dorsal protocerebrum, the deutocerebrum, the ventral nerve cords, as well as an elaborate fiber network within the brain, the ventral nerve cords and the peripheral nervous system including the heart nerve. Interestingly, we found that both peptides are co-expressed in all immunoreactive structures of *E. rowelli*, although their respective levels of expression differ at least in some cells and tissues. We were able to recognize three major patterns: (i) equal staining intensities of both peptides, (ii) higher Er-PDF-I signal, and (iii) higher Er-PDF-II signal. These different patterns were particularly evident in specific groups of somata within the brain (Figure 13).

Currently, we can only speculate about the functional significance of these observations. One possible reason for the differential expression of Er-PDF-I and Er-PDF-II might be their different functionality, as evidenced by our BRET assays with the deorphanized PDF receptor. Interestingly, the nematode *C. elegans* also possesses two *pdf* genes (albeit three PDF isoforms), which are co-expressed in certain cells including interneurons, and chemosensory and motor neurons (42). The PDF peptides in these cells seem to have opposing effects on locomotion, as PDF-1 deletion mutants and animals with overexpressed PDF-2 show the same atypical locomotory behavior (41, 49). Irrespective of whether or not similar opposing effects of the two peptides also exist in onychophorans, their co-expression in all immunoreactive cells of *E. rowelli* and at least in some cells of *C. elegans* suggests that this feature might have been present in the last common ancestor of Ecdysozoa.

The co-localization of the two PDF peptides in onychophorans and nematodes contrasts with what is known from arthropods that exhibit more than one PDF/PDH isoform such as decapod crustaceans. These animals express two to three PDH peptides (isoforms of either α-PDH or β-PDH), which are assumed to fulfill different functions based on their distinct localization (11, 43, 44, 46–48, 95–100). For example, while β-PDH II is expressed in the sinus gland of *Cancer productus*, and therefore most likely acts as a neurohormone, β-PDH is localized in the eyestalk, thus serving as a neuromodulator or neurotransmitter (11). It must be noted, however, that all crustacean PDH isoforms are derivatives and in-paralogs of *pdf-I* (Figure 1), whereas onychophorans and nematodes most likely inherited two *pdf* genes, *pdf-I* and *pdf-II* (*pdf-1* and *pdf-2 sensu* 41), from the last common ancestor of Ecdysozoa (38). From the evolutionary point of view, the multiple isoforms of decapod crustaceans are a derived feature of this group.

### Immunolocalization of the Onychophoran PDF Receptor Indicates Different Signal Transduction Mechanisms and Supports Hormonal Control

Localization of the deorphanized PDF receptor in *E. rowelli* using a specific Er-PDFR antibody revealed prominent immunoreactivity associated with membranes of specific cells in different organ systems. In contrast to the two PDF peptides, PDFR is not expressed in the ventral nerve cords but only in the brain and the connecting cords (101) within the central nervous system. There are three types of PDFR-expressing cells in the brain including those (i) co-expressing PDFR and PDF-I/PDF-II, (ii) not co-expressing but directly adjacent to PDF-I/PDF-II-expressing cells, and (iii) not closely associated with PDF-I/PDF-II immunoreactive cells. This indicates three different mechanisms of signal transduction:

- Co-localization of PDFR and the two PDF peptides suggests autoreception, i.e., feedback on its own cell, which has also been reported from fruit flies and Madeira cockroaches (34, 102). In insects, however, the majority of PDF-expressing cells are autoreceptive and they are typically outnumbered by PDFR-expressing cells (60), whereas PDF-immunoreactive cells are more numerous than PDFR-expressing cells in the onychophoran brain.
- In cells that do not co-express the PDF peptides and their receptor, the signal transduction might be accomplished through wiring transmission via the synaptic contacts or membrane juxtapositions of cells with direct contact to each other (103).
- Finally, cells that are not directly associated with each other might communicate via the hormonal release of peptides (i.e., volume transmission), like in decapod crustaceans (43, 95). The PDF-ir cells of onychophorans are indeed characterized by numerous varicosities and axonal terminals, which are reminiscent of typical release sites (38).

The so-called hypocerebral organs–enigmatic, vesicle-like structures associated with the onychophoran brain–have been repeatedly proposed to play a neurosecretory role (104, 105). Although we cannot rule out this potential function, the lack of PDF-I, PDF-II, and PDFR immunoreactivity in their tissues suggests that the hypocerebral organs do not produce or secrete PDF peptides; neither do they seem to be involved in the PDF/PDFR system.

Apart from the central nervous system, prominent PDFR signal is associated with the visual system of *E. rowelli*. In particular, membranes surrounding the microvilli of the photoreceptor cell processes (rhabdoms) and the pigment granules of the supportive cells [cf. (106)] are highly immunoreactive. PDH has been described to induce retinal pigment dispersion in the eyes of crustaceans under bright light conditions (1, 3, 5, 6). Whether or not such pigment dispersion occurs in the onychophoran eye is unclear, but it seems unlikely due to the generally low resolution of vision in these animals (107) and the position of pigment granules at the base of the rhabdomeric layer (106, 108, 109). Regardless of in which direction the pigment granules would move within the supportive cells, their basal position prevents shading/protection of photoreceptor processes from incident light. Additional PDFR immunoreactivity within the eye occurs in a few somata of the optic ganglion [cf. (109)]. Whether this signal is associated with glial cells, as reported from *D. melanogaster* (61), remains to be clarified.

Most intriguingly, besides the nervous and visual systems of *E. rowelli*, we detected PDFR in membranes of specific hemocytes. These hemocytes appeared blueish under the bright-field microscope and were localized in different parts of the circulatory system including the heart. Up to five major types of hemocytes have been described from onychophorans based on their ultrastructure (110, 111), but beyond this only little is known about their possible functions. At this point, we can only speculate that the blue-pigmented granules might be due to storage of hemocyanin (112) or, alternatively, might be associated with a role of these cells in immune defense, as hemocytes have been proposed to absorb dead ectodermal cells (113), which in *E. rowelli* contain blue pigment granules. Irrespective of whether these hemocytes are involved in the potential storage of hemocyanin or innate immune response, each of these roles might be controlled by the PDF/PDFR system depending on the amount of PDF peptides released into the hemolyph. This potentially novel function of PDF peptides associated with hemocytes requires further investigation. Nonetheless, the demonstrated occurrence of PDF receptor in cells of the visual and circulatory systems of *E. rowelli* clearly supports the suggested (38) hormonal role of PDF peptides in onychophorans.

## Conclusions

In this study, we deorphanized and immunolocalized the onychophoran PDF receptor and performed double labeling using specific antibodies against the two onychophoran PDF peptides. We further explored potential differences in the stimulation of PDFR by each peptide, revealing that PDF-II has a tenfold higher potency than PDF-I as an activating ligand. Double immunolabeling demonstrates that both onychophoran PDF peptides are co-expressed but their respective levels of expression show cell-specific variation. For example, some neurons express the same amount of both peptides, while others exhibit higher levels of either PDF-I or PDF-II. Whether this variation is due to cyclic changes, as for example in insects (33, 114, 115), remains to be clarified.

The detection of the onychophoran PDF receptor in cells that additionally express the two PDF peptides suggests autoreception, whereas spatial separation of PDFR- and PDF-expressing cells confirms hormonal release into the hemolymph (38). Hence, the PDF peptides of onychophorans might play a dual role—as hormones and as neurotransmitters or neuromodulators—similar to the PDH peptides of decapod crustaceans (7–12). Whether the PDF-releasing cells of onychophorans are light-responsive ultradian and circadian oscillators, as for example in the Madeira cockroach (116–119), is unknown. Future studies should therefore focus on clarifying whether there are any cycling patterns in the expression of the two peptides. Establishment of cell cultures (e.g., for *in vivo* calcium imaging) and corresponding behavioral assays would contribute to a better understanding of the PDF/PDFR system in Onychophora and the last common ancestors of Panarthropoda and Ecdysozoa.

## Data Availability Statement

The raw data supporting the conclusions of this article will be made available by the authors, without undue reservation, to any qualified researcher.

## Author Contributions

LH, CM, AW, FH, MS, and GM designed the research. LH conducted the BRET experiments and the phylogenetic analyses. CM, NM, and SH performed the immunolabeling experiments. NM, SH, SK, and SF carried out the specificity tests. CM, LH, NM, SH, and GM analyzed the data and wrote the first draft. All authors have read and approved the final manuscript.

## Conflict of Interest

The authors declare that the research was conducted in the absence of any commercial or financial relationships that could be construed as a potential conflict of interest.

## References

[1] Helfrich-Förster C Neuropeptide PDF plays multiple roles in the circadian clock of *Drosophila melanogaster*. Sleep Biol Rhythms. (2009) 7:130–43. 10.1111/j.1479-8425.2009.00408.x

[2] Meelkop E Temmerman L Schoofs L Janssen T. Signalling through pigment dispersing hormone-like peptides in invertebrates. Prog Neurobiol. (2011) 93:125–47. 10.1016/j.pneurobio.2010.10.004 21040756

[3] Larimer JL Smith JT Circadian rhythm of retinal sensitivity in crayfish: modulation by the cerebral and optic ganglia. J Comp Physiol. (1980) 136:313–26. 10.1007/BF00657351

[4] Rao KR Riehm JP. Pigment-dispersing hormones: a novel family of neuropeptides from arthropods. Peptides. (1988) 9:153–9. 10.1016/0196-9781(88)90239-2 2856640

[5] Verde M Barriga-Montoya C Fuentes-Pardo B. Pigment dispersing hormone generates a circadian response to light in the crayfish, *Procambarus clarkii*. Comp Biochem Phys A. (2007) 147:983–92. 10.1016/j.cbpa.2007.03.004 17428715

[6] Strauss J Dircksen H. Circadian clocks in crustaceans: identified neuronal and cellular systems. Front Biosci. (2010) 15:1040–74. 10.2741/3661 20515741

[7] Dircksen H Zahnow CA Gaus G Keller R Rao KR Riehm JP The ultrastructure of nerve endings containing pigment-dispersing hormone (PDH) in crustacean sinus glands: identification by an antiserum against a synthetic PDH. Cell Tissue Res. (1987) 250:377–87. 10.1007/BF00219082

[8] Mangerich S Keller R Dircksen H Rao RK Riehm JP Immunocytochemical localization of pigment-dispersing hormone (PDH) and its coexistence with FMRFamide-immunoreactive material in the eyestalks of the decapod crustaceans *Carcinus maenas* and *Orconectes limosus*. Cell Tissue Res. (1987) 250:365–75. 10.1007/BF00219081

[9] Mangerich S Keller R. Localization of pigment-dispersing hormone (PDH) immunoreactivity in the central nervous system of *Carcinus maenas* and *Orconectes limosus* (Crustacea), with reference to FMRFamide immunoreactivity in *O. limosus*. Cell Tissue Res. (1988) 253:199–208. 10.1007/BF00221755 3416337

[10] Nussbaum T Dircksen H Neuronal pathways of classical crustacean neurohormones in the central nervous system of the woodlouse, *Oniscus asellus* (L.). Philos Trans R Soc Lond B Biol Sci. (1995) 347:139–54. 10.1098/rstb.1995.0018

[11] Hsu Y-WA Stemmler EA Messinger DI Dickinson PS Christie AE De La Iglesia HO. Cloning and differential expression of two β-pigment-dispersing hormone (β-PDH) isoforms in the crab *Cancer productus*: evidence for authentic β-PDH as a local neurotransmitter and β-PDH II as a humoral factor. J Comp Neurol. (2008) 508:197–211. 10.1002/cne.21659 18311785

[12] Harzsch S Dircksen H Beltz BS. Development of pigment-dispersing hormone-immunoreactive neurons in the *American lobster*: homology to the insect circadian pacemaker system? Cell Tissue Res. (2009) 335:417–29. 10.1007/s00441-008-0728-z 19034522 PMC3072782

[13] Rao KR Mohrherr CJ Riehm JP Zahnow CA Norton S Johnson L. Primary structure of an analog of crustacean pigment-dispersing hormone from the lubber grasshopper *Romalea microptera*. J Biol Chem. (1987) 262:2672–5. 3818616

[14] Homberg U Würden S Dircksen H Rao KR Comparative anatomy of pigment-dispersing hormone-immunoreactive neurons in the brain of orthopteroid insects. Cell Tissue Res. (1991) 266:343–57. 10.1007/bf00318190

[15] Nässel DR Shiga S Wikstrand EM Rao RK. Pigment-dispersing hormone-immunoreactive neurons and their relation to serotonergic neurons in the blowfly and cockroach visual system. Cell Tissue Res. (1991) 266:511–23. 10.1007/BF00318593 1811881

[16] Nässel DR Shiga S Mohrherr CJ Rao KR. Pigment-dispersing hormone-like peptide in the nervous system of the flies *Phormia* and *Drosophila*: immunocytochemistry and partial characterization. J Comp Neurol. (1993) 331:183–98. 10.1002/cne.903310204 8509499

[17] Helfrich-Förster C Homberg U Pigment-dispersing hormone-immunoreactive neurons in the nervous system of wild-type *Drosophila melanogaster* and of several mutants wih altered circadian rhythmicity. J Comp Neurol. (1993) 337:177–90. 10.1002/cne.903370202 8276996

[18] Stengl M Homberg U. Pigment-dispersing hormone-immunoreactive neurons in the cockroach *Leucophaea maderae* share properties with circadian pacemaker neurons. J Comp Physiol A. (1994) 175:203–13. 10.1007/bf00215116 8071895

[19] Petri B Stengl M Würden S Homberg U. Immunocytochemical characterization of the accessory medulla in the cockroach *Leucophaea maderae*. Cell Tissue Res. (1995) 282:3–19. 10.1007/BF00319128 8581923

[20] Würden S Homberg U. Immunocytochemical mapping of serotonin and neuropeptides in the accessory medulla of the locust, *Schistocerca gregaria*. J Comp Neurol. (1995) 362:305–19. 10.1002/cne.903620302 8576441

[21] Reischig T Stengl M Morphology and pigment-dispersing hormone immunocytochemistry of the accessory medulla, the presumptive circadian pacemaker of the cockroach *Leucophaea maderae*: a light-and electron-microscopic study. Cell Tissue Res. (1996) 285:305–19. 10.1007/s004410050648

[22] Reischig T Stengl M. Optic lobe commissures in a three-dimensional brain model of the cockroach *Leucophaea maderae*: a search for the circadian coupling pathways. J Comp Neurol. (2002) 443:388–400. 10.1002/cne.10133 11807846

[23] Reischig T Stengl M. Ultrastructure of pigment-dispersing hormone-immunoreactive neurons in a three-dimensional model of the accessory medulla of the cockroach *Leucophaea maderae*. Cell Tissue Res. (2003) 314:421–35. 10.1007/s00441-003-0772-7 14557869

[24] Petri B Stengl M. Pigment-dispersing hormone shifts the phase of the circadian pacemaker of the cockroach *Leucophaea maderae*. J Neurosci. (1997) 17:4087–93. 10.1523/JNEUROSCI.17-11-04087.1997 9151725 PMC6573569

[25] Helfrich-Förster C Stengl M Homberg U. Organization of the circadian system in insects. Chronobiol Int. (1998) 15:567–94. 10.3109/07420529808993195 9844747

[26] Renn SCP Park JH Rosbash M Hall JC Taghert PH. A *pdf* neuropeptide gene mutation and ablation of PDF neurons each cause severe abnormalities of behavioral circadian rhythms in *Drosophila*. Cell. (1999) 99:791–802. 10.1016/s0092-8674(00)81676-1 10619432

[27] Persson MGS Eklund MB Dircksen H Muren JE Nässel DR. Pigment-dispersing factor in the locust abdominal ganglia may have roles as circulating neurohormone and central neuromodulator. J Neurobiol. (2001) 48:19–41. 10.1002/neu.1040 11391647

[28] Bloch G Solomon SM Robinson GE Fahrbach SE. Patterns of PERIOD and pigment-dispersing hormone immunoreactivity in the brain of the European honeybee (*Apis mellifera*): age- and time-related plasticity. J Comp Neurol. (2003) 464:269–84. 10.1002/cne.10778 12900924

[29] Sehadová H Sauman I Sehnal F. Immunocytochemical distribution of pigment-dispersing hormone in the cephalic ganglia of polyneopteran insects. Cell Tissue Res. (2003) 312:113–25. 10.1007/s00441-003-0705-5 12712321

[30] Reischig T Petri B Stengl M. Pigment-dispersing hormone (PDH)-immunoreactive neurons form a direct coupling pathway between the bilaterally symmetric circadian pacemakers of the cockroach *Leucophaea maderae*. Cell Tissue Res. (2004) 318:553–64. 10.1007/s00441-004-0927-1 15578273

[31] Wei H Yasar H Funk NW Giese M Baz E-S Stengl M. Signaling of pigment-dispersing factor (PDF) in the Madeira cockroach *Rhyparobia maderae*. PLoS ONE. (2014) 9:e108757. 10.1371/journal.pone.0108757 25269074 PMC4182629

[32] Stengl M Arendt A. Peptidergic circadian clock circuits in the Madeira cockroach. Curr Opin Neurobiol. (2016) 41:44–52. 10.1016/j.conb.2016.07.010 27575405

[33] Beer K Kolbe E Kahana NB Yayon N Weiss R Menegazzi P. Pigment-dispersing factor-expressing neurons convey circadian information in the honey bee brain. Open Biol. (2018) 8:170224. 10.1098/rsob.170224 29321240 PMC5795053

[34] Gestrich J Giese M Shen W Zhang Y Voss A Popov C. Sensitivity to pigment-dispersing factor (PDF) is cell-type specific among PDF-expressing circadian clock neurons in the madeira cockroach. J Biol Rhythms. (2018) 33:35–51. 10.1177/0748730417739471 29179611

[35] Vosko AM Schroeder A Loh DH Colwell CS. Vasoactive intestinal peptide and the mammalian circadian system. Gen Comp Endocrinol. (2007) 152:165–75. 10.1016/j.ygcen.2007.04.018 17572414 PMC1994114

[36] Veenstra JA. Neurohormones and neuropeptides encoded by the genome of *Lottia gigantea*, with reference to other mollusks and insects. Gen Comp Endocrinol. (2010) 167:86–103. 10.1016/j.ygcen.2010.02.010 20171220

[37] Veenstra JA. Neuropeptide evolution: neurohormones and neuropeptides predicted from the genomes of *Capitella teleta* and *Helobdella robusta*. Gen Comp Endocrinol. (2011) 171:160–75. 10.1016/j.ygcen.2011.01.005 21241702

[38] Mayer G Hering L Stosch JM Stevenson PA Dircksen H. Evolution of pigment-dispersing factor neuropeptides in Panarthropoda: insights from Onychophora (velvet worms) and Tardigrada (water bears). J Comp Neurol. (2015) 523:1865–85. 10.1002/cne.23767 25722044

[39] Semmens DC Mirabeau O Moghul I Pancholi MR Wurm Y Elphick MR. Transcriptomic identification of starfish neuropeptide precursors yields new insights into neuropeptide evolution. Open Biol. (2016) 6:150224. 10.1098/rsob.150224 26865025 PMC4772807

[40] Giribet G Edgecombe GD. Current understanding of Ecdysozoa and its internal phylogenetic relationships. Integr Comp Biol. (2017) 57:455–66. 10.1093/icb/icx072 28957525

[41] Janssen T Husson SJ Lindemans M Mertens I Rademakers S Donck KV. Functional characterization of three G protein-coupled receptors for pigment dispersing factors in *Caenorhabditis elegans*. J Biol Chem. (2008) 283:15241–9. 10.1074/jbc.M709060200 18390545 PMC3258896

[42] Janssen T Husson SJ Meelkop E Temmerman L Lindemans M Verstraelen K. Discovery and characterization of a conserved pigment dispersing factor-like neuropeptide pathway in *Caenorhabditis elegans*. J Neurochem. (2009) 111:228–41. 10.1111/j.1471-4159.2009.06323.x 19686386

[43] Rao KR Riehm JP. Pigment-dispersing hormones. Ann N Y Acad Sci. (1993) 680:78–88. 10.1111/j.1749-6632.1993.tb19676.x 8512238

[44] Klein JM Mohrherr CJ Sleutels F Riehm JP Rao KR. Molecular cloning of two pigment-dispersing hormone (PDH) precursors in the blue crab *Callinectes sapidus* reveals a novel member of the PDH neuropeptide family. Biochem Biophys Res Commun. (1994) 205:410–6. 10.1006/bbrc.1994.2680 7999056

[45] Desmoucelles-Carette C Sellos D Van Worhmoudt A. Molecular cloning of the pigment dispersing hormone in a crustacean. Ann N Y Acad Sci. (1998) 839:395–6. 10.1111/j.1749-6632.1998.tb10810.x 9629186

[46] Yang W-J Aida K Nagasawa H. Characterization of chromatophorotropic neuropeptides from the kuruma prawn *Penaeus japonicus*. Gen Comp Endocrinol. (1999) 114:415–24. 10.1006/gcen.1999.7266 10336829

[47] Ohira T Nagasawa H Aida K. Molecular cloning of cDNAs encoding two pigment-dispersing hormones and two corresponding genes from the kuruma prawn (*Penaeus japonicus*). Mar Biotechnol. (2002) 4:463–70. 10.1007/s10126-002-0042-9 14961239

[48] Ohira T Tsutsui N Kawazoe I Wilder MN. Isolation and characterization of two pigment-dispersing hormones from the whiteleg shrimp, *Litopenaeus vannamei*. Zool Sci. (2006) 23:601–6. 10.2108/zsj.23.601 16908959

[49] Herrero A Romanowski A Meelkop E Caldart C Schoofs L Golombek DA. Pigment-dispersing factor signaling in the circadian system of *Caenorhabditis elegans*. Genes Brain Behav. (2015) 14:493–501. 10.1111/gbb.12231 26113231

[50] Barrios A Ghosh R Fang C Emmons SW Barr MM PDF-1 neuropeptide signaling modulates a neural circuit for mate-searching behavior in *C. elegans Nat Neurosci*. (2012) 15:1675–82. 10.7554/eLife.36547.001 PMC3509246 23143519

[51] Sato S Chuman Y Matsushima A Tominaga Y Shimohigashi Y Shimohigashi M. A circadian neuropeptide, pigment-dispersing factor-PDF, in the last-summer cicada *Meimuna opalifera*: cDNA cloning and immunocytochemistry. Zool Sci. (2002) 19:821–8. 10.2108/zsj.19.821 12193798

[52] Honda T Matsushima A Sumida K Chuman Y Sakaguchi K Onoue H. Structural isoforms of the circadian neuropeptide PDF expressed in the optic lobes of the cricket *Gryllus bimaculatus*: immunocytochemical evidence from specific monoclonal antibodies. J Comp Neurol. (2006) 499:404–21. 10.1002/cne.21112 16998911

[53] Wen C-J Lee H-J. Mapping the cellular network of the circadian clock in two cockroach species. Arch Insect Biochem Physiol. (2008) 68:215–31. 10.1002/arch.20236 18618766

[54] Závodská R Wen C-J Hrdý I Sauman I Lee H-J Sehnal F. Distribution of corazonin and pigment-dispersing factor in the cephalic ganglia of termites. Arthropod Struct Dev. (2008) 37:273–86. 10.1016/j.asd.2008.01.005 18394958

[55] Wei H El Jundi B Homberg U Stengl M. Implementation of pigment-dispersing factor-immunoreactive neurons in a standardized atlas of the brain of the cockroach *Leucophaea maderae*. J Comp Neurol. (2010) 518:4113–33. 10.1002/cne.22471 20878779

[56] Ikeno T Numata H Goto SG Shiga S. Involvement of the brain region containing pigment-dispersing factor-immunoreactive neurons in the photoperiodic response of the bean bug, *Riptortus pedestris*. J Exp Biol. (2014) 217:453–62. 10.1242/jeb.091801 24198258

[57] Mertens I Vandingenen A Johnson EC Shafer OT Li W Trigg JS. PDF receptor signaling in *Drosophila* contributes to both circadian and geotactic behaviors. Neuron. (2005) 48:213–9. 10.1016/j.neuron.2005.09.009 16242402

[58] Hyun S Lee Y Hong S-T Bang S Paik D Kang J. *Drosophila* GPCR Han is a receptor for the circadian clock neuropeptide PDF. Neuron. (2005) 48:267–78. 10.1016/j.neuron.2005.08.025 16242407

[59] Lear BC Merrill CE Lin J-M Schroeder A Zhang L Allada R. A G protein-coupled receptor, *groom-of-PDF*, is required for PDF neuron action in circadian behavior. Neuron. (2005) 48:221–7. 10.1016/j.neuron.2005.09.008 16242403

[60] Shafer OT Kim DJ Dunbar-Yaffe R Nikolaev VO Lohse MJ Taghert PH. Widespread receptivity to neuropeptide PDF throughout the neuronal circadian clock network of *Drosophila* revealed by real-time cyclic AMP imaging. Neuron. (2008) 58:223–37. 10.1016/j.neuron.2008.02.018 18439407 PMC2586874

[61] Im SH Taghert PH. PDF receptor expression reveals direct interactions between circadian oscillators in *Drosophila*. J Comp Neurol. (2010) 518:1925–45. 10.1002/cne.22311 20394051 PMC2881544

[62] Hering L Henze MJ Kohler M Kelber A Bleidorn C Leschke M. Opsins in Onychophora (velvet worms) suggest a single origin and subsequent diversification of visual pigments in arthropods. Mol Biol Evol. (2012) 29:3451–8. 10.1093/molbev/mss148 22683812

[63] Reid AL Review of the Peripatopsidae (Onychophora) in Australia, with comments on peripatopsid relationships. Invertebr. Taxon. (1996) 10:663–936. 10.1071/IT9960663

[64] Baer A Mayer G. Comparative anatomy of slime glands in Onychophora (velvet worms). J Morphol. (2012) 273:1079–88. 10.1002/jmor.20044 22707384

[65] I5k C The i5K Initiative: advancing arthropod genomics for knowledge, human health, agriculture, and the environment. J Hered. (2013) 104:595–600. 10.1093/jhered/est050 23940263 PMC4046820

[66] Thomas GWC Dohmen E Hughes DST Murali SC Poelchau M Glastad K. Gene content evolution in the arthropods. Genome Biol. (2020) 21:15. 10.1186/s13059-019-1925-7 31969194 PMC6977273

[67] Martin M Cutadapt removes adapter sequences from high-throughput sequencing reads. EMBnet J. (2011) 17:10–2. 10.14806/ej.17.1.200

[68] Smeds L Künstner A. ConDeTri-a content dependent read trimmer for Illumina data. PLoS ONE. (2011) 6: e26314. 10.1371/journal.pone.0026314 22039460 PMC3198461

[69] Peng Y Leung HC Yiu S-M Lv M-J Zhu X-G Chin FY. IDBA-tran: a more robust de novo de Bruijn graph assembler for transcriptomes with uneven expression levels. Bioinformatics. (2013) 29:i326–34. 10.1093/bioinformatics/btt219 23813001 PMC3694675

[70] Altschul SF Madden TL Schäffer AA Zhang J Zhang Z Miller W. Gapped BLAST and PSI-BLAST: a new generation of protein database search programs. Nucleic Acids Res. (1997) 25:3389–402. 10.1093/nar/25.17.3389 9254694 PMC146917

[71] Pándy-Szekeres G Munk C Tsonkov TM Mordalski S Harpsøe K Hauser AS. GPCRdb in 2018: adding GPCR structure models and ligands. Nucleic Acids Res. (2018) 46:D440–6. 10.1093/nar/gkx1109 29155946 PMC5753179

[72] Frickey T Lupas A. CLANS: a Java application for visualizing protein families based on pairwise similarity. Bioinformatics. (2004) 20:3702–4. 10.1093/bioinformatics/bth444 15284097

[73] Koski LB Golding GB The closest BLAST hit is often not the nearest neighbor. J. Mol. Evol. (2001) 52:540–2. 10.1007/s002390010184 11443357

[74] Hoare SR. Mechanisms of peptide and nonpeptide ligand binding to class B G-protein-coupled receptors. Drug Discov Today. (2005) 10:417–27. 10.1016/S1359-6446(05)03370-2 15808821

[75] Hollenstein K De Graaf C Bortolato A Wang M-W Marshall FH Stevens RC. Insights into the structure of class B GPCRs. Trends Pharmacol Sci. (2014) 35:12–22. 10.1016/j.tips.2013.11.001 24359917 PMC3931419

[76] Sonnhammer EL Eddy SR Durbin R. Pfam: a comprehensive database of protein domain families based on seed alignments. Proteins. (1997) 28:405–20. 10.1002/(SICI)1097-0134(199707)28:3<405::AID-PROT10>3.0.CO;2-L 9223186

[77] Finn RD Coggill P Eberhardt RY Eddy SR Mistry J Mitchell AL. The Pfam protein families database: towards a more sustainable future. Nucleic Acids Res. (2016) 44:D279–85. 10.1093/nar/gkv1344 26673716 PMC4702930

[78] Katoh K Rozewicki J Yamada KD. MAFFT online service: multiple sequence alignment, interactive sequence choice and visualization. Brief Bioinform. (2017) 20:1160–6. 10.1093/bib/bbx108 28968734 PMC6781576

[79] Dress AWM Flamm C Fritzsch G Grünewald S Kruspe M Prohaska SJ. Noisy: identification of problematic columns in multiple sequence alignments. Algorithm Mol Biol. (2008) 3:7. 10.1186/1748-7188-3-7 18577231 PMC2464588

[80] Stamatakis A. RAxML version 8: a tool for phylogenetic analysis and post-analysis of large phylogenies. Bioinformatics. (2014) 30:1312–3. 10.1093/bioinformatics/btu033 24451623 PMC3998144

[81] Letunic I Bork P. Interactive Tree Of Life v2: online annotation and display of phylogenetic trees made easy. Nucleic Acids Res. (2011) 39:W475–8. 10.1093/nar/gkr201 21470960 PMC3125724

[82] Treffkorn S Kahnke L Hering L Mayer G. Expression of NK cluster genes in the onychophoran *Euperipatoides rowelli*: implications for the evolution of NK family genes in nephrozoans. EvoDevo. (2018) 9:17. 10.1186/s13227-018-0105-2 30026904 PMC6050708

[83] Nikolaev VO Bünemann M Hein L Hannawacker A Lohse MJ. Novel single chain cAMP sensors for receptor-induced signal propagation. J Biol Chem. (2004) 279:37215–8. 10.1074/jbc.C400302200 15231839

[84] Ponsioen B Zhao J Riedl J Zwartkruis F Van Der Krogt G Zaccolo M. Detecting cAMP-induced Epac activation by fluorescence resonance energy transfer: Epac as a novel cAMP indicator. EMBO Rep. (2004) 5:1176–80. 10.1038/sj.embor.7400290 15550931 PMC1299185

[85] Klarenbeek J Goedhart J Van Batenburg A Groenewald D Jalink K. Fourth-generation Epac-based FRET sensors for cAMP feature exceptional brightness, photostability and dynamic range: characterization of dedicated sensors for FLIM, for ratiometry and with high affinity. PLoS ONE. (2015) 10:e0122513. 10.1371/journal.pone.0122513 25875503 PMC4397040

[86] Stefanini M De Martino C Zamboni L. Fixation of ejaculated spermatozoa for electron microscopy. Nature. (1967) 216:173–4. 10.1038/216173a0 4862079

[87] Robson E Lockwood A Ralph R Composition of the blood in Onychophora. Nature. (1966) 209:533 10.1038/209533a05919597

[88] Schindelin J Arganda-Carreras I Frise E Kaynig V Longair M Pietzsch T. Fiji: an open-source platform for biological-image analysis. Nat Meth. (2012) 9:676–82. 10.1038/nmeth.2019 22743772 PMC3855844

[89] Schneider CA Rasband WS Eliceiri KW. NIH Image to ImageJ: 25 years of image analysis. Nat Meth. (2012) 9:671–5. 10.1038/nmeth.2089 22930834 PMC5554542

[90] Prinz A Diskar M Herberg FW. Application of bioluminescence resonance energy transfer (BRET) for biomolecular interaction studies. ChemBioChem. (2006) 7:1007–12. 10.1002/cbic.200600048 16755626

[91] Schulze J Neupert S Schmidt L Predel R Lamkemeyer T Homberg U. Myoinhibitory peptides in the brain of the cockroach *Leucophaea maderae* and colocalization with pigment-dispersing factor in circadian pacemaker cells. J Comp Neurol. (2012) 520:1078–97. 10.1002/cne.22785 22095637

[92] Nery LEM De Lauro Castrucci AM. Pigment cell signalling for physiological color change. Comp Biochem Physiol A. (1997) 118:1135–44. 10.1016/S0300-9629(97)00045-5 9505423

[93] De Rooij J Zwartkruis FJT Verheijen MHG Cool RH Nijman SMB Wittinghofer A. Epac is a Rap1 guanine-nucleotide-exchange factor directly activated by cyclic AMP. Nature. (1998) 396:474–7. 10.1038/24884 9853756

[94] Nikolaev VO Lohse MJ. Monitoring of cAMP synthesis and degradation in living cells. Physiology. (2006) 21:86–92. 10.1152/physiol.00057.2005 16565474

[95] Rao KR Riehm JP The pigment-dispersing hormone family: chemistry, structure-activity relations, and distribution. Biol Bull. (1989) 177:225–9. 10.2307/1541937

[96] De Kleijn DPV Linck B Klein JM Weidemann WM Keller R Van Herp F. Structure and localization of mRNA encoding a pigment dispersing hormone (PDH) in the eyestalk of the crayfish *Orconectes limosus*. FEBS Lett. (1993) 321:251–5. 10.1016/0014-5793(93)80119-f 8477858

[97] Desmoucelles-Carette C Sellos D Van Wormhoudt A. Molecular cloning of the precursors of pigment dispersing hormone in crustaceans. Biochem Biophys Res Commun. (1996) 221:739–43. 10.1006/bbrc.1996.0666 8630031

[98] Rao KR Crustacean pigmentary-effector hormones: chemistry and functions of RPCH, PDH, and related peptides. Am Zool. (2001) 41:364–79. 10.1093/icb/41.3.364

[99] Bulau P Meisen I Schmitz T Keller R Peter-Katalinić J. Identification of neuropeptides from the sinus gland of the crayfish *Orconectes limosus* using nanoscale on-line liquid chromatography tandem mass spectrometry. Mol Cell Proteomics. (2004) 3:558–64. 10.1074/mcp.M300076-MCP200 14981133

[100] Fu Q Goy MF Li L. Identification of neuropeptides from the decapod crustacean sinus glands using nanoscale liquid chromatography tandem mass spectrometry. Biochem Biophys Res Commun. (2005) 337:765–78. 10.1016/j.bbrc.2005.09.111 16214114

[101] Martin C Gross V Hering L Tepper B Jahn H De Sena Oliveira I. The nervous and visual systems of onychophorans and tardigrades: learning about arthropod evolution from their closest relatives. J Comp Physiol A. (2017) 203:565–90. 10.1007/s00359-017-1186-4 28600600

[102] Taghert PH Nitabach MN. Peptide neuromodulation in invertebrate model systems. Neuron. (2012) 76:82–97. 10.1016/j.neuron.2012.08.035 23040808 PMC3466441

[103] Zoli M Jansson A Syková E Agnati LF Fuxe K. Volume transmission in the CNS and its relevance for neuropsychopharmacology. Trends Pharmacol Sci. (1999) 20:142–50. 10.1016/S0165-6147(99)01343-7 10322499

[104] Sanchez S Cellules neurosécrétrices et organes infracérébraux de *Peripatopsis moseleyi* Wood (Onychophores) et neurosécrétion chez *Nymphon gracile* Leach (Pycnogonides). Arch Zool Exp Gen. (1958) 96:57–62.

[105] Eriksson BJ Tait NN Norman JM Budd GE An ultrastructural investigation of the hypocerebral organ of the adult *Euperipatoides kanangrensis* (Onychophora, Peripatopsidae). Arthropod Struct Dev. (2005) 34:407–18. 10.1016/j.asd.2005.03.002

[106] Eakin RM Westfall JA. Fine structure of the eye of peripatus (Onychophora). Z Zellforsch Mikrosk Anat. (1965) 68:278–300. 10.1007/BF00342434 5869902

[107] Kirwan JD Graf J Smolka J Mayer G Henze MJ Nilsson DE Low–resolution vision in a velvet worm (Onychophora). J Exp Biol. (2018) 221:jeb175802 10.1242/jeb.175802 29626113

[108] Dakin WJ The eye of peripatus. Q J Microsc Sci. (1921) 65:163–72.

[109] Mayer G. Structure and development of onychophoran eyes—what is the ancestral visual organ in arthropods? Arthropod Struct Dev. (2006) 35:231–45. 10.1016/j.asd.2006.06.003 18089073

[110] Lavallard R Campiglia S Contribution a l'hêmatologie de *Peripatus acacioi* Marcus et Marcus (Onychophora) — I. Structure et ultrastructure des hêmocytes. Ann Sci Nat Zool Biol Anim. (1975) 17:67–92.

[111] Ravindranath MH Onychophorans and myriapods. In: Ratcliffe NA, Rowley AF, editors. Invertebrate Blood Cells: Arthropods to Urochordates, Invertebrates and Vertebrates Compared. London: Academic Press (1981). p. 329–52.

[112] Kusche K Ruhberg H Burmester T. A hemocyanin from the Onychophora and the emergence of respiratory proteins. Proc Natl Acad Sci USA. (2002) 99:10545–8. 10.1073/pnas.152241199 12149441 PMC124969

[113] Manton SM Studies on the Onychophora, IV. The passage of spermatozoa into the ovary in Peripatopsis and the early development of the ova. Philos Trans R Soc B Biol Sci. (1938) 228:421–44. 10.1098/rstb.1938.0001

[114] Park JH Helfrich-Förster C Lee G Liu L Rosbash M Hall JC. Differential regulation of circadian pacemaker output by separate clock genes in *Drosophila*. Proc Natl Acad Sci USA. (2000) 97:3608–13. 10.1073/pnas.97.7.3608 10725392 PMC16287

[115] Vafopoulou X Terry KL Steel CGH. The circadian timing system in the brain of the fifth larval instar of *Rhodnius prolixus* (hemiptera). J Comp Neurol. (2010) 518:1264–82. 10.1002/cne.22274 20151359

[116] Schneider N-L Stengl M. Pigment-dispersing factor and GABA synchronize cells of the isolated circadian clock of the cockroach *Leucophaea maderae*. J Neurosci. (2005) 25:5138–47. 10.1523/jneurosci.5138-a-04.2005 15917454 PMC6724822

[117] Schneider N-L Stengl M. Extracellular long-term recordings of the isolated accessory medulla, the circadian pacemaker center of the cockroach *Leucophaea maderae*, reveal ultradian and hint circadian rhythms. J Comp Physiol A. (2007) 193:35–42. 10.1007/s00359-006-0169-7 16983545

[118] Wei H Stengl M. Light affects the branching pattern of peptidergic circadian pacemaker neurons in the brain of the cockroach Leucophaea maderae. J Biol Rhythms. (2011) 26:507–17. 10.1177/0748730411419968 22215609

[119] Wei H Stengl M Ca2+-dependent ion channels underlying spontaneous activity in insect circadian pacemaker neurons. Eur J Neurosci. (2012) 36:3021–9. 10.1111/j.1460-9568.2012.08227.x 22817403

