# Supplementary material for: Analysis of Pigment-Dispersing Factor Neuropeptides and Their Receptor in a Velvet Worm
Source: Front Endocrinol (Lausanne). 2020 May 12;11:273. doi: 10.3389/fendo.2020.00273 (PMC7235175; doi:10.3389/fendo.2020.00273)
Supplement: Supplementary file 3 [file Image_3.pdf]

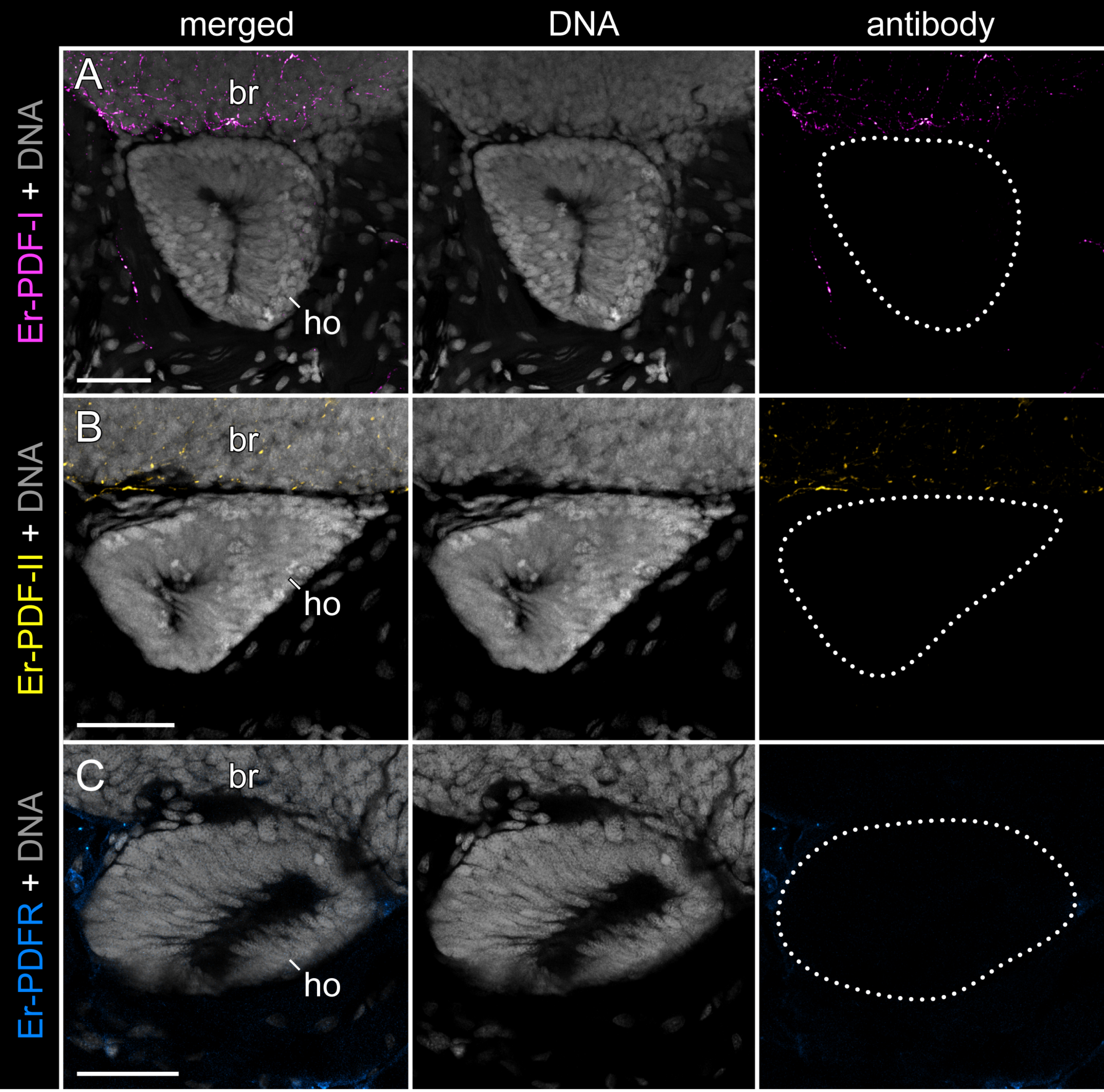

Supplementary Figure 3 Immunolocalization of Er-PDF-I, Er-PDF-II and Er-PDFR in the hypocerebral organs of *E. rowelli*. Confocal laser scanning micrographs of vibratome sections. Er-PDF-I (magenta), Er-PDF-II (yellow); Er-PDFR (cyan) and DNA (grey). Dorsal is up in all images. **(A)** Er-PDF-I, **(B)** Er-PDF-II and **(C)** Er-PDFR immunoreactivities are not seen in the hypocerebral organs (indicated by dotted line in right column). br, brain; ho, hypocerebral organs. Scale bars: 50 µm.
