# Supplementary material for: Analysis of Pigment-Dispersing Factor Neuropeptides and Their Receptor in a Velvet Worm
Source: Front Endocrinol (Lausanne). 2020 May 12;11:273. doi: 10.3389/fendo.2020.00273 (PMC7235175; doi:10.3389/fendo.2020.00273)
Supplement: Supplementary file 4 [file Image_4.pdf]

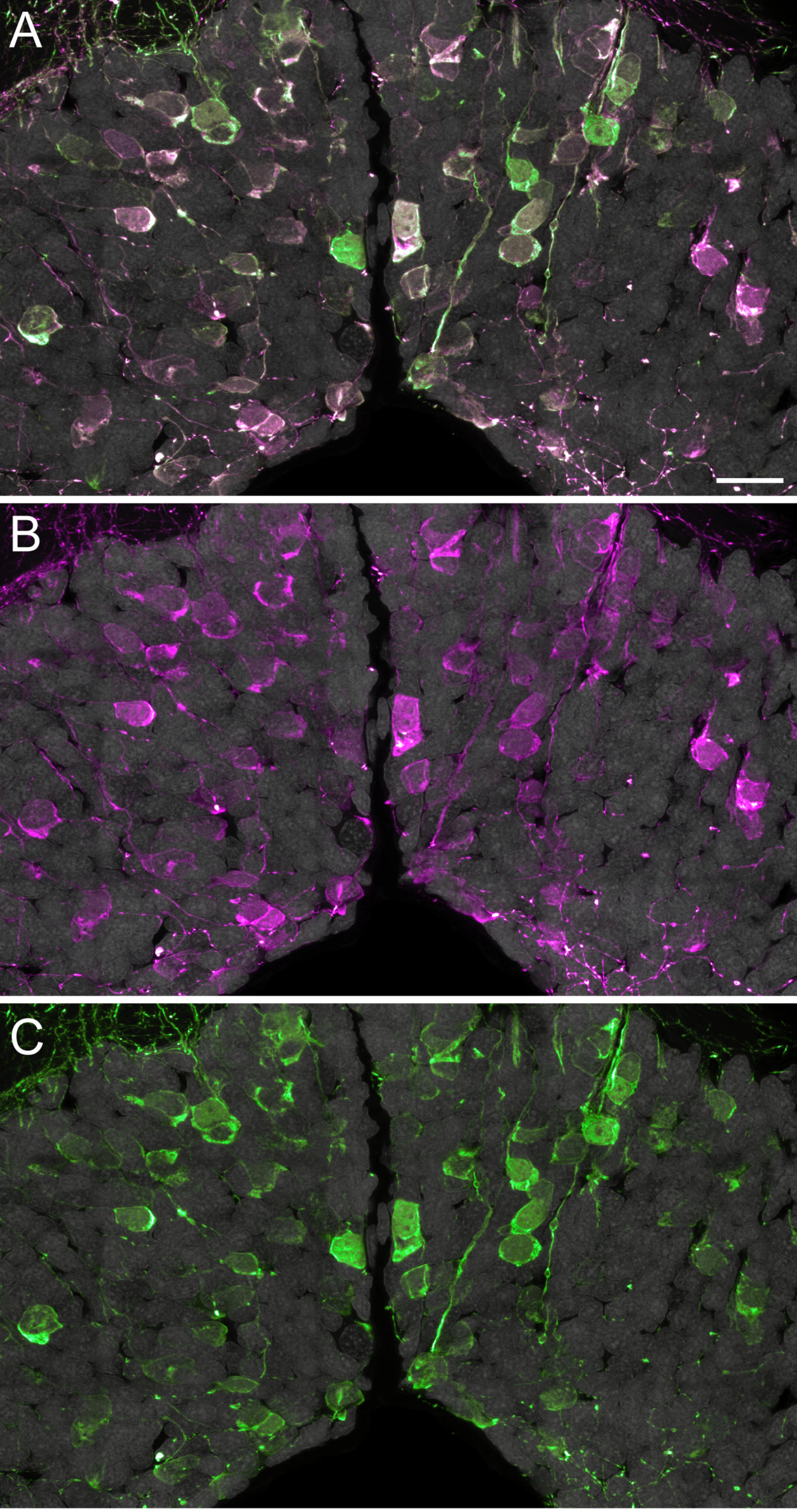

Supplementary Figure 4 Combined immunolocalization of Er-PDF-I and Er-PDF-II in *E. rowelli*. Confocal laser scanning micrographs of vibratome sections. Dorsal is up in all images. Er-PDF-I (magenta), Er-PDF-II (green), and DNA (grey). **(A–C)** Same sample of ventral perikaryal layer showing Er-PDF-I-ir and Er-PDF-II-ir **(A)**, Er-PDF-I-ir **(B)** and Er-PDF-II-ir **(C)**. Note co-localization of Er-PDF-I and Er-PDF-II in all immunoreactive somata, albeit at different levels. Scale bar: 20 μm.
