# Supplementary material for: Analysis of Pigment-Dispersing Factor Neuropeptides and Their Receptor in a Velvet Worm
Source: Front Endocrinol (Lausanne). 2020 May 12;11:273. doi: 10.3389/fendo.2020.00273 (PMC7235175; doi:10.3389/fendo.2020.00273)
Supplement: Supplementary file 5 [file Image_5.pdf]

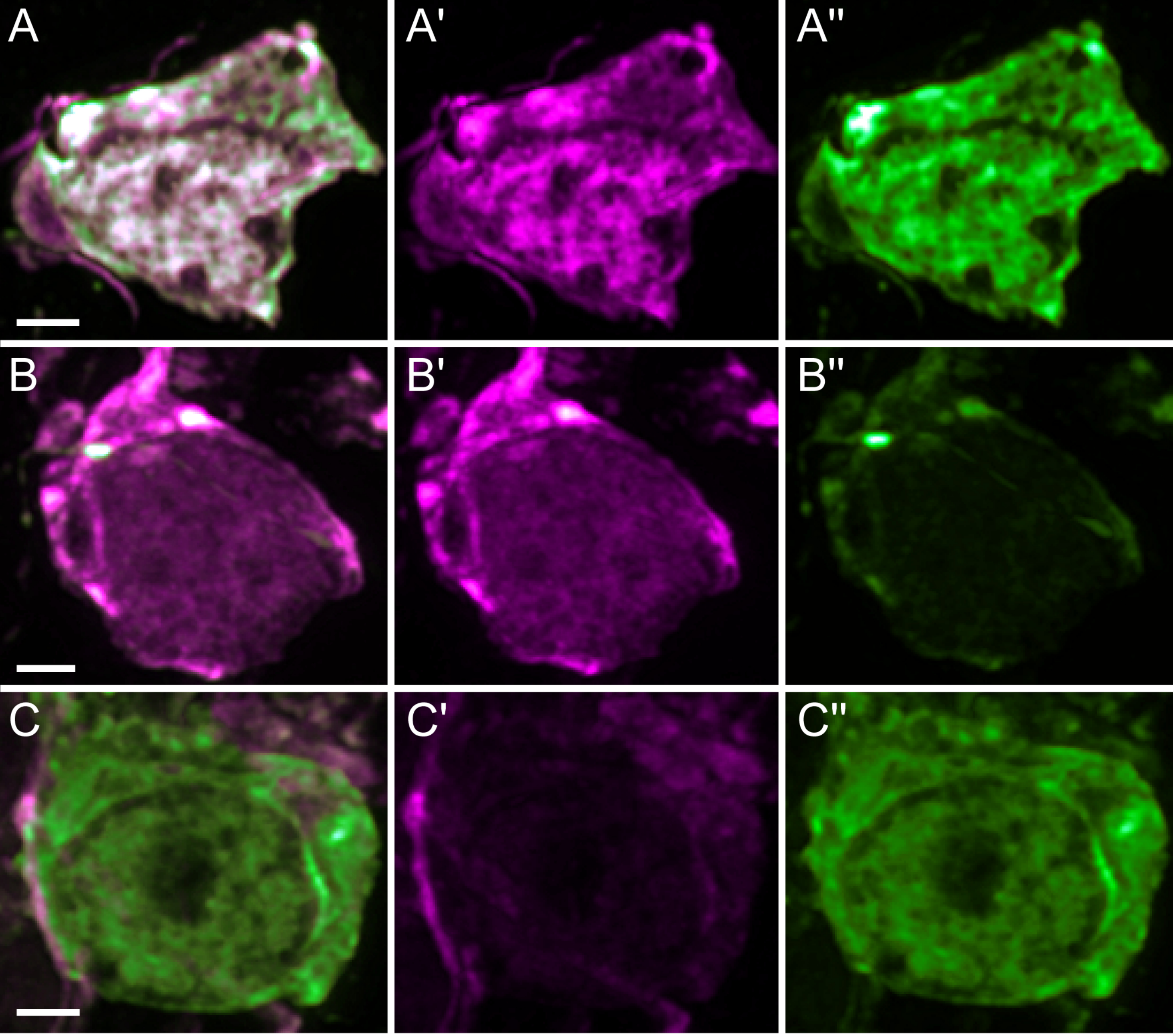

Supplementary Figure 5 Combined immunolocalization of Er-PDF-I and Er-PDF-II in single somata of *E. rowelli*. Confocal laser scanning micrographs of vibratome sections. Er-PDF-I (magenta) and Er-PDF-II (green). Somata occur in three variants: **(A)** Er-PDF-I and Er-PDF-II at equal levels, **(B)** Er-PDF-I-ir at a higher level, and **(C)** Er-PDF-II-ir at a higher level. Scale bars: 2 µm.
