# Supplementary material for: Analysis of Pigment-Dispersing Factor Neuropeptides and Their Receptor in a Velvet Worm
Source: Front Endocrinol (Lausanne). 2020 May 12;11:273. doi: 10.3389/fendo.2020.00273 (PMC7235175; doi:10.3389/fendo.2020.00273)
Supplement: Supplementary file 6 [file Image_6.pdf]

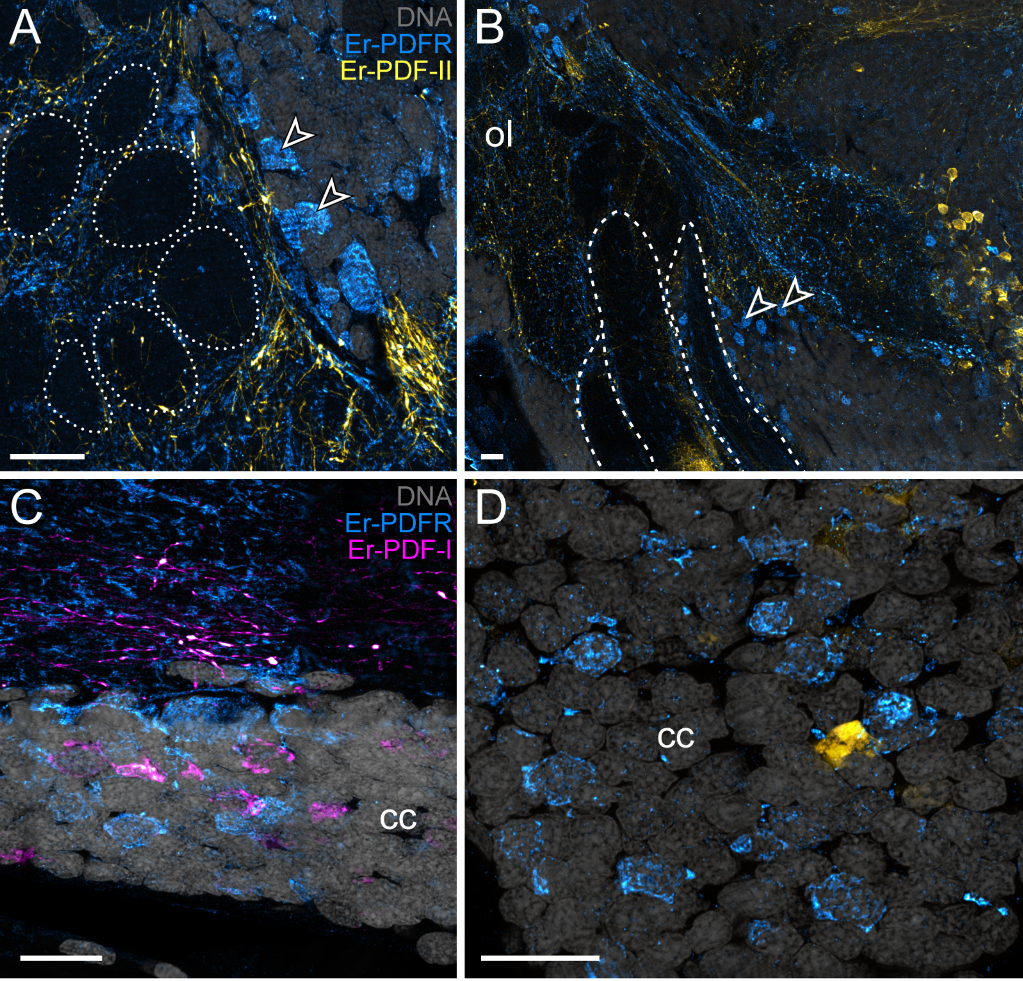

Supplementary Figure 6 Combined immunolocalization of Er-PDF-I and Er-PDF-I in *E. rowelli*. Confocal laser scanning micrographs of vibratome sections. Dorsal is up in all images. Er-PDF-I (magenta), Er-PDF-II (yellow), Er-PDFR (cyan) and DNA (grey). PDFR-ir somata are located in close proximity to olfactory glomeruli (dotted lines) **(A)** and mushroom body lobes (dashed lines) **(B)**. **(C)** Additional group of PDFR-ir somata in connecting cords **(C,D)**. cc, connecting cord; ol, olfactory lobe. Scale bars: 20 µm.
