# Supplementary material for: Analysis of Pigment-Dispersing Factor Neuropeptides and Their Receptor in a Velvet Worm
Source: Front Endocrinol (Lausanne). 2020 May 12;11:273. doi: 10.3389/fendo.2020.00273 (PMC7235175; doi:10.3389/fendo.2020.00273)
Supplement: Supplementary file 7 [file Image_7.pdf]

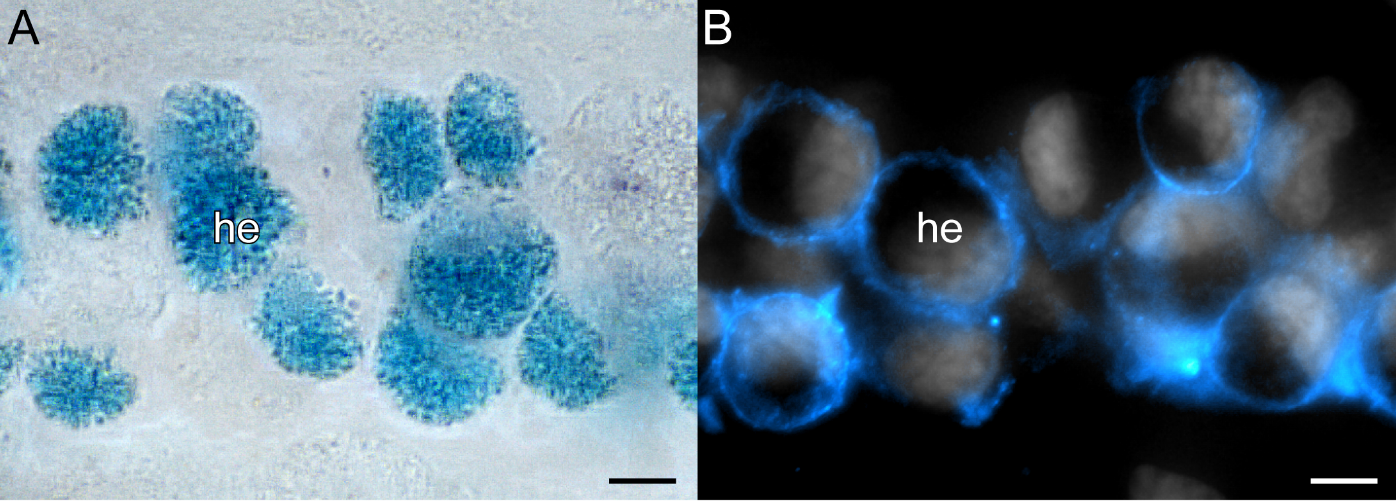

Supplementary Figure 7 Immunolocalization of Er-PDFR in hemocytes of *E. rowelli*. **(A)** Transmitted light and **(B)** fluorescence light micrographs of the same region. Er-PDFR (cyan) and DNA (grey). **(A)** Blue granulated pigments occur in a subset of hemocytes in heart lumen. **(B)** PDFR immunoreactivity in membrane of same hemocytes. he, hemocyte. Scale bars: 5 µm.
