# Supplementary material for: Analysis of Pigment-Dispersing Factor Neuropeptides and Their Receptor in a Velvet Worm
Source: Front Endocrinol (Lausanne). 2020 May 12;11:273. doi: 10.3389/fendo.2020.00273 (PMC7235175; doi:10.3389/fendo.2020.00273)
Supplement: Supplementary file 1 [file Image_1.pdf]

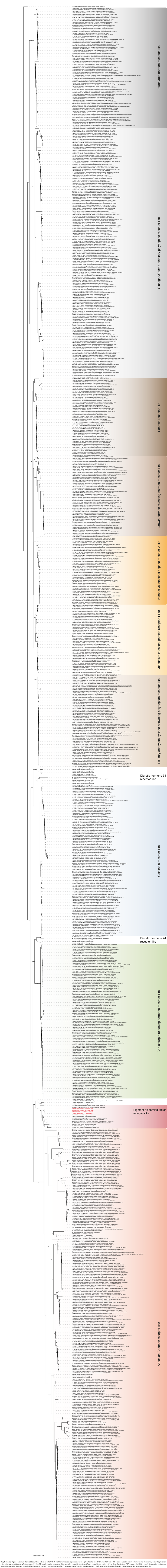

Supplementary Figure 1 Maximum likelihood tree under a dataset-specific GTR+G model of amino acid sequence evolution (log likelihood score: -87,233.49) of 993 class B G protein-coupled receptors obtained from a cluster analysis and the placement of the putative pigment-dispersing factor receptor (PDFR) of *E. rowelli*. Note the occurrence of the respective transcripts from three different transcriptomes of *E. rowelli* in a clade of protostome PDF receptors (highlighted in red). Note also the fractioning in two contigs (transcript 10426 and transcript 39667) observed in one of the transcriptomes (ER10). Bootstrap values (from 100 pseudoreplicates) > 50 % are given at the nodes. Scale bar indicates the number of substitutions per site. .
